# Effectiveness and Safety of Under or Over-dosing of Direct Oral Anticoagulants in Atrial Fibrillation: A Systematic Review and Meta-analysis of 148909 Patients From 10 Real-World Studies

**DOI:** 10.3389/fphar.2021.645479

**Published:** 2021-03-18

**Authors:** Nan-Nan Shen, Chi Zhang, Na Wang, Jia-Liang Wang, Zhi-Chun Gu, Hua Han

**Affiliations:** ^1^ Department of Pharmacy, Affiliated Hospital of Shaoxing University, Shao Xing, China; ^2^ Department of Pharmacy, Renji Hospital, School of Medicine, Shanghai Jiaotong University, Shanghai, China; ^3^ Department of Pharmacy, The Second Affiliated Hospital of Chongqing Medical University, Chongqing, China; ^4^ School of Medicine, Tongji University, Shanghai, China

**Keywords:** atrial fibrillation, direct oral anticoagulants, stroke, bleeding, real-world, under-dose, over-dose

## Abstract

**Background:** In routine clinical practice, non-standard doses of direct oral anticoagulants (DOACs) are commonly used in patients with atrial fibrillation (AF). However, data on the clinical outcomes of non-standard doses of DOACs are limited.

**Methods:** The MEDLINE, Embase, and Cochrane Library databases were systematically searched from their inception until 30 June 2020 for studies that reported the effectiveness or safety outcomes of non-standard doses of DOACs compared with on-label doses of DOACs in patients with atrial fibrillation. Non-standard doses of DOACs were defined as under or over-dose of DOACs based on the recommended standard doses in drug labels. A random-effects meta-analysis was performed to calculate the pooled hazard ratio and associated 95% confidence interval (95% confidence interval). Subgroup analyses were conducted according to individual DOACs and different geographic regions.

**Results:** Ten articles involving 148,909 patients with AF were included. There were no significant differences between under-dosing and on-label dosing with respect to stroke/systematic embolism (HR: 1.01, 95% CI: 0.93–1.09), major bleeding (HR: 0.98, 95% CI: 0.77–1.19), intracranial haemorrhage (HR: 1.07, 95% CI: 0.74–1.40), gastrointestinal bleeding (HR: 1.10, 95% CI: 0.82–1.39), and myocardial infarction (HR: 1.07, 95% CI: 0.89–1.25), except for an increased risk of death (HR: 1.37, 95% CI: 1.01–1.73). We observed a significant association between over-dosing of DOACs and increased risk of stroke/systematic embolism (HR: 1.18, 95% CI: 1.04–1.32), major bleeding (HR: 1.16, 95% CI: 1.03–1.29), and death (HR: 1.21, 95% CI: 1.03–1.38) compared with on-label dosing. Furthermore, over-dosing of DOACs increased the risk of stroke/systematic embolism (HR: 1.16; 95% CI: 1.00–1.33) and major bleeding events (HR: 1.18; 95% CI: 1.00–1.37) in Asian patients.

**Conclusion:** A reduced dose of DOACs might be safely and effectively used in clinical practice, especially in Asian patients, whereas high-dose DOACs might not be well tolerated by Asian patients.

## Introduction

Treatments to prevent stroke, especially oral anticoagulant use, are crucial for the management of patients with atrial fibrillation (AF). Randomised controlled trials (RCTs) have confirmed the non-inferiority of direct oral anticoagulants (DOACs) in stroke prevention compared with vitamin K antagonists (VKAs), with a lower bleeding risk (Connolly et al., 2009; Granger et al., 2011; Patel et al., 2011; Giugliano et al., 2013). Accordingly, DOACs, including dabigatran, rivaroxaban, apixaban, and edoxaban, have been rapidly and massively adopted as therapy for patients with AF, especially for initial users (Huiart et al., 2018). It is notable that some patients with AF should be treated with dose-adjusted DOACs approved by the United States. Food and Drug Administration (FDA) according to the characteristics of patients (e.g., age, body weight, and renal function) and concomitantly used medications (Camm et al., 2012; Lehr et al., 2012; January et al., 2014; Steinberg et al., 2016; Martin et al., 2018; De Caterina et al., 2019). Nevertheless, non-standard doses of DOACs are commonly used in clinical practice for patients with AF who should be administered standard doses according to the drug instructions (Okumura et al., 2017; Lee et al., 2019), with a prevalence of 26.2–39.6% (Murata et al., 2019; Yu et al., 2020). In clinical practice, clinicians tend to prescribe inappropriate under- or over-dose of DOACs owing to the following: over-considering the bleeding risk and neglecting dose adjustment, especially in Asian clinicians (Son et al., 2018). Therefore, studies have now focused on the clinical outcomes of non-standard dosing of DOACs. A previous United States national registry study including 5738 AF patients treated with DOACs reported that over-dosing of DOACs was closely related to increased all-cause mortality, whereas under-dosing was associated with increased cardiovascular disease-related hospitalisation (Steinberg et al., 2016). However, another registry study conducted in Japan reported similar stroke/systemic embolism (SE) and death events in patients with AF administered standard doses and under-doses of DOACs, and higher composite events (stroke/SE, major bleeding, or death) in patients administered over-doses than in those administered standard doses of DOACs (Murata et al., 2019). Apparently, there are substantial differences among studies in terms of the clinical outcomes associated with non-standard doses of DOACs. Therefore, the effectiveness and safety of non-standard doses of DOACs in patients with AF remain unclear, and high-quality relevant evidence in this regard is limited. To fill this knowledge gap, all available evidence was collected and summarised to conduct a comprehensive and rigorous systematic review of the clinical outcomes of non-standard dosing of DOACs in patients with AF.

## Methods

This systematic review and meta-analysis was performed based on the PRISMA reporting guidelines and Cochrane Collaboration (Stroup et al., 2000; Liberati et al., 2009). The protocol was prospectively registered in PROSPERO (CRD42020170600).

### Data Sources and Searches

The MEDLINE, Embase, and Cochrane Library databases were searched from their inception to 30 June 2020 for relevant studies, and the language was restricted to English. The detailed search strategy is presented in (Supplementary Table S1). In addition, the references cited in all retrieved articles and relevant reviews were manually searched to identify additional studies.

### Study Selection and Outcomes

Studies were included according to the following criteria: 1) prospective or retrospective cohort studies of patients with AF and 2) reported effectiveness or safety outcomes of non-standard dosing of DOACs (dabigatran, rivaroxaban, apixaban, and edoxaban). Valvular AF patients or patients receiving short-term DOACs after catheter ablation were excluded. Studies that only reported crude data or those published in the form of conference abstracts or letters were also excluded. If the same data source or overlapping data were reported in more than one study, the most comprehensive data with the longest follow-up period were included. On-label dose of a DOAC was defined as the dose received in accordance with the drug instructions. Non-standard dose of a DOAC was classified as under- and over-dosing, despite the patient meeting the standard dose criteria. The primary outcomes of effectiveness were stroke/SE, death, and myocardial infarction (MI). The primary safety outcomes were major bleeding, intracranial haemorrhage (ICH), and gastrointestinal (GI) bleeding. To determine eligibility, two authors (N. S. and C. Z.) independently reviewed all study titles and abstracts, and entire papers were assessed based on entry criteria. Disagreements between the reviewers were resolved by consensus or discussion with the corresponding investigator (Z.C. and H. H.).

### Data Extraction

Two authors (N. S. and C. Z.) independently extracted the following data from each included article: study characteristics (the first author and publication year, country or region, data source, study design, follow-up period, proportion of each DOAC in the study, total patient number, and definition of DOAC non-standard dosing), demographics and clinical characteristics (mean age, sex ratio, comorbidities, concomitant medications, CHA_2_DS_2_-VASc score, and HAS-BLED score), and effectiveness and safety outcomes. The geographic regions of the included studies were classified as Asia, North America, and Europe.

### Quality Assessment

The methodological quality of all included studies was evaluated using the modified Newcastle-Ottawa Scale (NOS), which involved five domains: sample population, sample size, participation rate, assessment of outcome, and analytical methods to control bias (Cota et al., 2013). Each item was assigned a maximum of 2 points, and a total score of > 6 points was considered high quality (Supplementary Table S2).

### Data Synthesis and Statistical Analysis

To compare the clinical outcomes of non-standard doses vs. on-label doses of DOACs in patients with AF, a random-effects model meta-analysis was conducted to calculate the pooled hazard ratio (HR) and 95% confidence interval (CI), regardless of the presence of heterogeneity. Heterogeneity among studies was evaluated using the *I* ^2^ value, with *I* ^2^ > 50% representing a high degree of heterogeneity. Subgroup analyses were conducted based on individual DOACs (dabigatran, rivaroxaban, apixaban, and edoxaban) and geographic regions (Asia, North America, and Europe). Interaction analyses (*P* for interaction) were performed to evaluate comparability in each subgroup. To explore the influence of each study on the synthetic outcomes of non-standard dosing of DOAC, a sensitivity analysis was performed by removing each study from the pool. A meta-regression analysis was performed to assess factors influencing the outcomes. Publication bias was explored qualitatively using funnel plots and quantitatively using Begg’s test and Egger’s test (Liberati et al., 2009). All statistical analyses were conducted using STATA version13.0 (Statacorp, College Station, Texas, TX, United States).

## Results

### Study Selection and Characteristics

**FIGURE 1:**
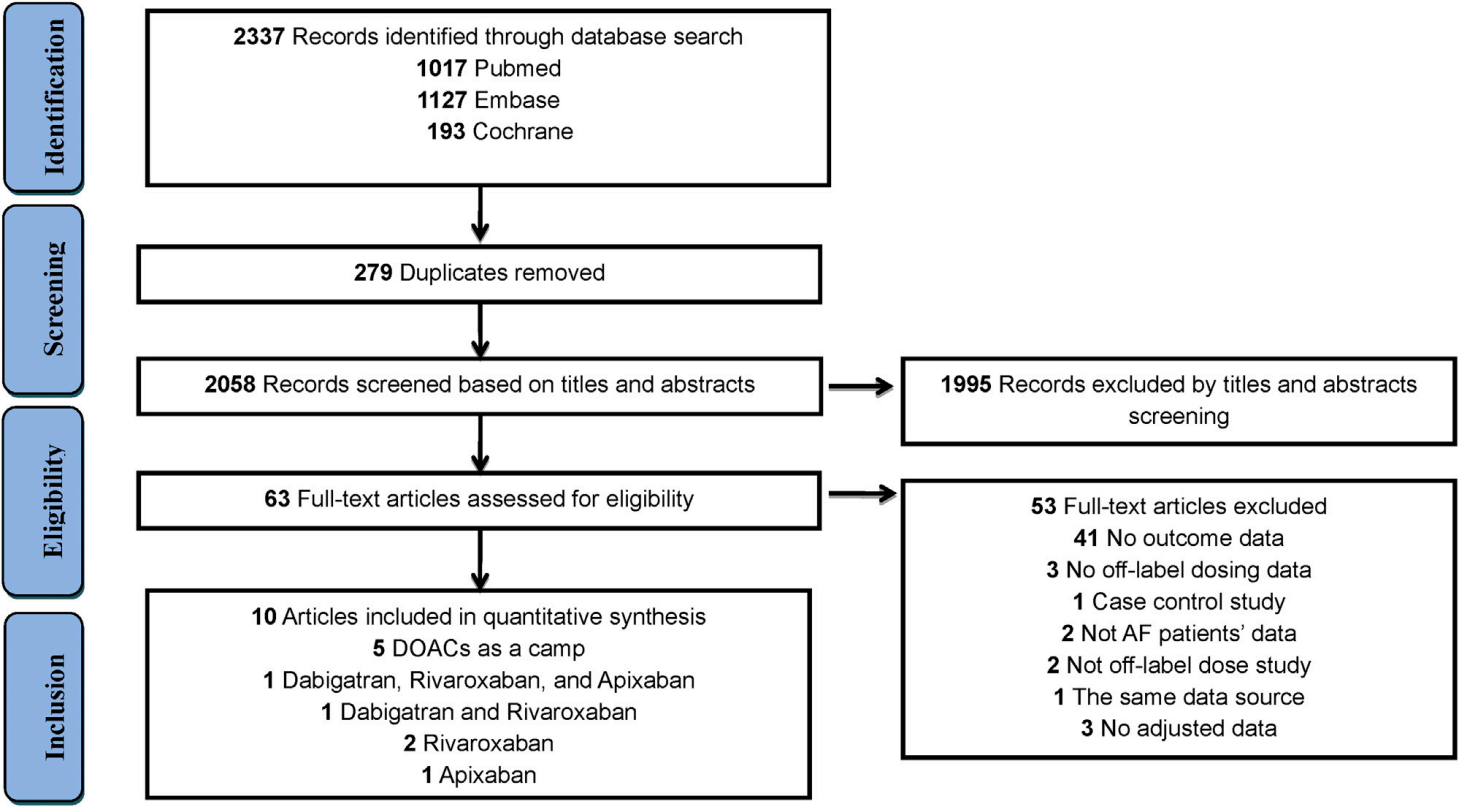
Flow diagram for the selection of eligible studies.

The study selection process is outlined in Figure 1. In total, 2,337 articles were identified through an initial literature search, after eliminating 279 duplicate studies. After screening titles and abstracts, 1,995 articles were excluded. Thereafter, 53 studies were removed in the full-text review process for the reasons outlined in (Supplementary Table S3). Finally, 10 studies (Steinberg et al., 2016; Yao et al., 2017; Arbel et al., 2019; Cheng et al., 2019; Ikeda et al., 2019; Murata et al., 2019; Briasoulis et al., 2020; de Almeida et al., 2020; Salameh et al., 2020; Yu et al., 2020) involving 148,909 patients were included in this study. Among these studies, DOACs were assessed as a camp in 5 studies, dabigatran in 2 studies, rivaroxaban in 3 studies, and apixaban in 2 studies (Figure 1). Detailed characteristics of all 10 studies are summarised in Table 1. Among the 10 studies, 3 studies were conducted in North America (all in the United States), 6 in Asia (1 in Taiwan, 2 in Japan, 1 in Korea, and 2 in Israel), and 1 in Europe (Portugal). The definition of non-standard dosing of DOACs and clinical outcomes in each included study are presented in (Supplementary Tables S4–S5).

**TABLE 1:** Detailed characteristics of the included studies.

Study	Study design	Country or region	Data source	Follow-up	DOACs proportion	Total number
Steinberg et al. (2016)	Prospective cohort study	USA	ORBIT-AF II I (outcomes registry for better informed treatment of atrial fibrillation phase II)	0.99 years	Dabigatran (7.4%); rivaroxaban (53.6%); apixaban (39%)	5738
Cheng et al. (2019)	Retrospective cohort study	Taiwan	Taipei veterans general hospital	2.23 years	Rivaroxaban (100%)	2214
Yao et al. (2017)	Retrospective cohort study	USA	OptumLabs data warehouse	3.6 months	Dabigatran (31.8%); rivaroxaban (43.2%); apixaban (25.0%)	14865
Murata et al. (2019)	Prospective cohort study	Japan	SAKURA AF registry	39.3 months	NR	1658
Arbel et al. (2019)	Retrospective cohort study	Israel	Clalit health services	23 months	NR	8425
Ikeda et al. (2019)	Prospective cohort study	Japan	XAPASS, a real-world Japanese prospective, single-arm, observational study.	1 year	Rivaroxaban (100%)	6521
Briasoulis et al. (2020)	Retrospective cohort study	USA	Medicare beneficiaries enrolled in a large United States health plan with prescription drug coverage.	15.1 months	Dabigatran (29.0%); rivaroxaban (71.0%)	27747
Salameh et al. (2020)	Retrospective cohort study	Israel	The computerized database of clalit health services (CHS)	60 months	Apixaban (100%)	27765
Yu et al. (2020)	Retrospective cohort study	Korea	Korean national health insurance service database	3 years	Dabigatran (30.5%); rivaroxaban (37.5%); apixaban (22.2%); and edoxaban (9.7%)	53649
de Almeida et al. (2020)	Retrospective cohort study	Portugal	The internal medicine department of coimbra university hospital	1 year	NR	327

AF, atrial fibrillation; USA, United States of America.

### Patient Characteristics and Quality Assessment

The detailed demographics and clinical characteristics of patients with AF in each included study are outlined in Supplementary Table S6. The mean age of patients was 72.8 years, and 45.1% of the patients were women. The mean CHA_2_DS_2_-VASc and HAS-BLED scores were 4.0 and 2.0, respectively. The mean body mass index was 27.5 kg/m^2^, and the use rate of concomitant antiplatelet agents was 25.7%. The main comorbidities were hypertension (83.4%), heart failure (39.1%), diabetes mellitus (36.8%), and transient ischaemic attack (18.7%). All included studies satisfied the following risk bias items: sample population, sample size, and participation rate. The overall quality of the included studies was generally high, and the NOS score ranged from 8 to 9 (Supplementary Table S7).

### Effectiveness and Safety of Under-dosing of DOACs and Subgroup Analyses

**FIGURE 2:**
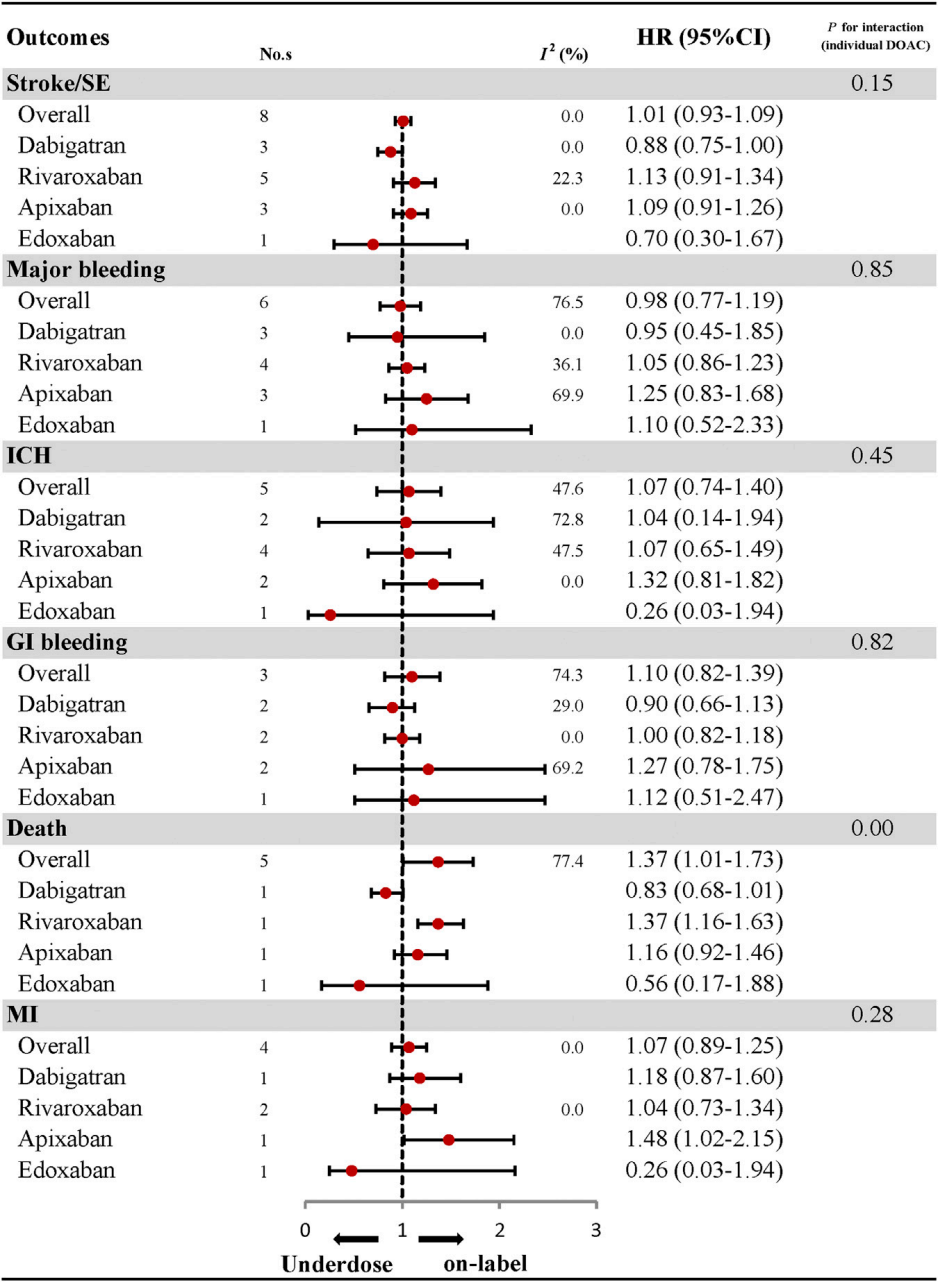
Effectiveness and safety of under-dose DOACs and individual DOAC. No., number of included studies; DOAC, direct oral anticoagulant; HR, hazard ratio; SE, systematic embolism; ICH, intracranial haemorrhage; GI bleeding, gastrointestinal bleeding; MI, myocardial infarction.

**FIGURE 3:**
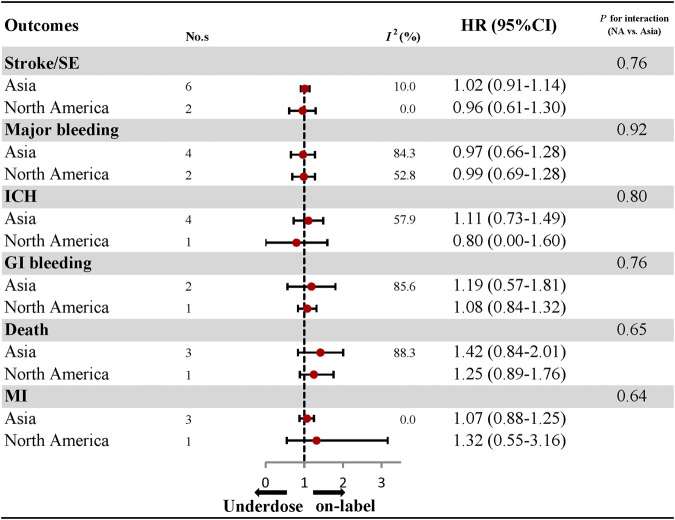
Effectiveness and safety of under-dose DOACs by regions. No., number of included studies; HR, hazard ratio; SE, systematic embolism; ICH, intracranial haemorrhage; GI bleeding, gastrointestinal bleeding; MI, myocardial infarction.

With respect to effectiveness outcomes, there was no significant difference between under-dosing and on-label dosing in terms of stroke/SE (HR: 1.01, 95% CI: 0.93–1.09, *I* ^*2*^: 0.0%) and MI (HR: 1.07, 95% CI: 0.89–1.25, *I* ^*2*^: 0.0%), with the exception of a higher rate of death (HR: 1.37, 95% CI: 1.01–1.73, *I* ^*2*^: 77.4%). Safety outcomes, including major bleeding (HR, 0.98; 95% CI, 0.77–1.19; *I* ^*2*^, 76.5%), ICH (HR: 1.07, 95% CI: 0.74–1.40, *I* ^*2*^: 47.6%), and GI bleeding (HR: 1.10, 95% CI: 0.82–1.39, *I* ^*2*^: 74.3%), were also similar between under-dosing and on-label dosing of DOACs (Figure 2; Supplementary Figures S1–S6). Analyses of the individual DOACs (dabigatran, rivaroxaban, apixaban, and edoxaban) are outlined in Figure 2; Supplementary Figures S7–S11. Under-dosing of each DOAC presented similar results for each effectiveness and safety outcome. Just one study was included to assess death associated with the under-dosing of rivaroxaban and MI with the under-dosing of apixaban, and found an increased risk (HR: 1.37, 95% CI: 1.16–1.63 for death; HR: 1.48, 95% CI: 1.02–2.15 for MI). The results for different geographic regions (Asia and North America) were also similar between under-dosing and on-label dosing (Figure 3; Supplementary Figures S12–S17). Statistical heterogeneity of effectiveness and safety outcomes was not detected in any subgroup analyses (*P*_interaction_ > 0.05 for each outcome).

### Effectiveness and Safety of Over-dosing of DOACs and Subgroup Analyses

**FIGURE 4:**
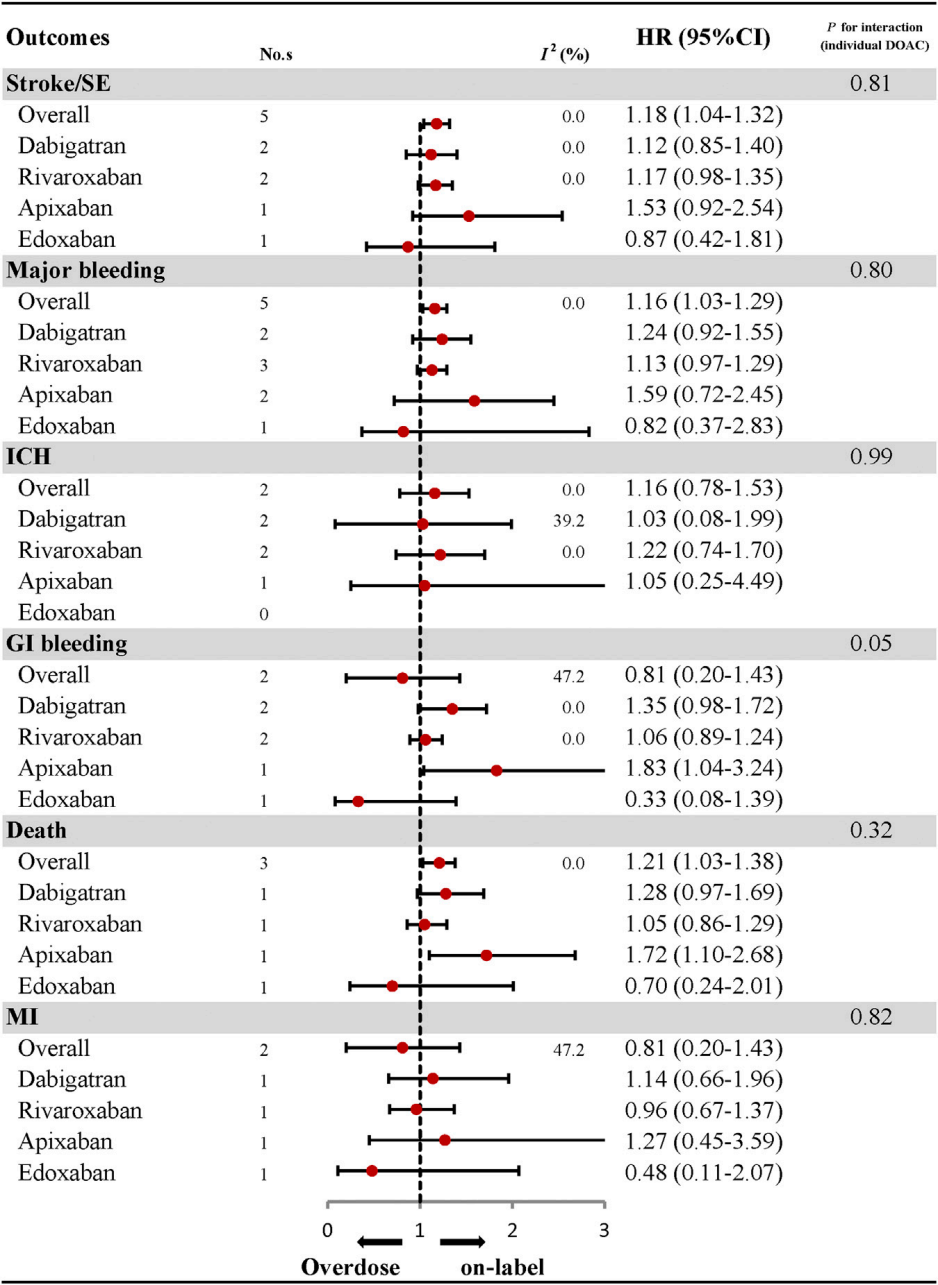
Effectiveness and safety of over-dose DOACs and individual DOAC.

**FIGURE 5:**
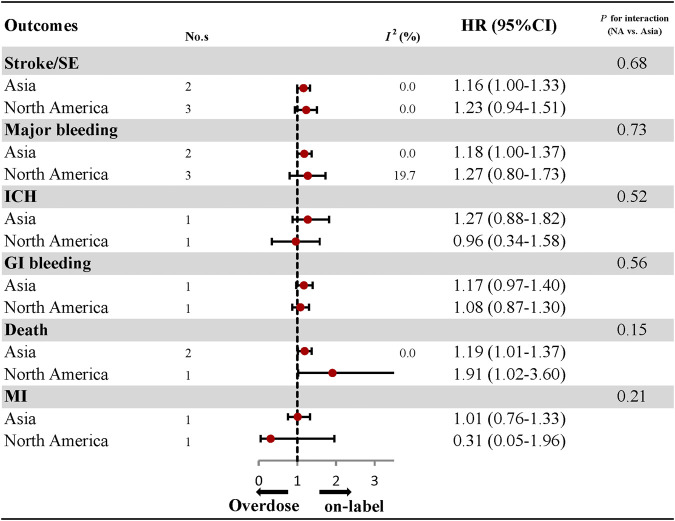
Effectiveness and safety of over-dose DOACs by regions.

The comparative results of total and individual DOACs between over-dosing and on-label dosing are presented in Figure 4; Supplementary Figures S18–S27. A significant association between over-dosing of DOACs and increased risk of stroke (HR: 1.18, 95% CI: 1.04–1.32, *I* ^*2*^: 0.0%), major bleeding (HR: 1.16, 95% CI: 1.03–1.29, *I* ^*2*^: 0.0%), and death (HR: 1.21, 95% CI: 1.03–1.38, *I* ^*2*^: 0.0%), was observed compared with on-label dosing. There were no significant differences in the outcomes of ICH (HR: 1.16, 95% CI: 0.78–1.53, *I* ^*2*^: 0.0%), GI bleeding (HR: 0.81, 95% CI: 0.20–1.43, *I* ^*2*^: 47.2%), and MI (HR: 0.81, 95% CI: 0.20–1.43, *I* ^*2*^: 47.2%) between over-dosing and on-label dosing of DOACs. Considering individual DOACs, similar results were observed between over-dosing and on-label dosing in all the outcomes evaluated. Among the different geographic regions, an increased risk of stroke (HR: 1.16, 95% CI: 1.00–1.33, *I* ^*2*^: 0.0%), major bleeding (HR: 1.18, 95% CI: 1.00–1.37, *I* ^*2*^: 0.0%), and death (HR: 1.19, 95% CI: 1.01–1.37, *I* ^*2*^: 0.0%) was observed in Asian patients. Other outcomes of ICH, GI bleeding, and MI were similar in both Asian and North American patients (Figure 5; Supplementary Figures S28–S30). No apparent heterogeneity was found in any of the subgroup analyses (*P*_interaction_ > 0.05 for each outcome).

### Sensitivity Analyses and Meta-regression

Analyses were repeated by sequentially removing each study, and the pooled results were consistent with the results of the main sensitivity analyses (Supplementary Tables S8–S9). Meta-regression analyses did not reveal any potential influencing factors associated with the clinical outcomes of non-standard doses of DOACs (*p* > 0.05 for each variable; Supplementary Tables S10, S11).

### Publication Bias

A visual inspection of the funnel plot showed a relative symmetry, suggesting that the publication bias was not a concern overall (Supplementary Tables S31, S32).

## Discussion

In this study, we comprehensively assessed the clinical outcomes of non-standard dosing of DOACs in patients with AF in a real-world setting. No significant difference was detected between under-dosing and on-label dosing of DOACs in terms of both effectiveness and safety outcomes. A significant association was observed between over-dosing of DOACs and increased risks of stroke/SE, major bleeding, and death compared with on-label dosing. Almost all clinical outcomes were similar between non-standard (under-dose and over-dose) and on-label dosing by different geographic regions.

As reported in previous studies, non-standard doses of DOACs are more likely to be prescribed to frail patients (Cheng et al., 2019; Lee et al., 2019). Patients treated with non-standard doses of DOACs, especially under-doses, are elderly individuals, more likely women, with a low body weight (≤60 kg), or a high CHA_2_DS_2_-VASc score compared with those treated with standard doses (Cheng et al., 2019; Lee et al., 2019). In addition, a history of renal dysfunction, stroke, and bleeding was also a risk factor for non-standard dosing of DOACs (Cheng et al., 2019; Lee et al., 2019). Some observational studies have focused on the clinical outcomes of non-standard doses of DOACs. A nationwide Danish study indicated that under-dosing of DOACs might increase the risk of stroke (Nielsen et al., 2017). Another United States prospective registry study suggested that over-dosing of DOACs was associated with a higher risk of mortality than on-label dosing (Steinberg et al., 2016). Whereas, a Japanese study have reported similar stroke/SE and death events in patients with AF administered standard doses and under-doses of DOACs and higher composite events in the over-dose group than in the standard dose group (Murata et al., 2019). Moreover, patient adherence of DOACs still remains a significant challenge in AF patients, which could definitely influence the outcomes (Maura et al., 2017). Therefore, findings regarding the effectiveness and safety of non-standard doses of DOACs are not consistent.

In our study, the effectiveness and safety outcomes were similar between under-dosing and on-label dosing of DOACs, and this is different from the results of increased stroke and death risks in under-dosed patients reported in previous studies (Steinberg et al., 2016; Nielsen et al., 2017). The difference in outcomes could be partially explained by several patient characteristics, especially body weight. Body weight could be a vital factor influencing the risk of stroke/SE and bleeding when patients are under DOAC therapy (Murata et al., 2019). It is suspected that patients with a higher body weight might require a higher dose of DOACs than patients with a lower body weight. Therefore, higher stroke/SE and death rates (Steinberg et al., 2016; Nielsen et al., 2017) were associated with under-doses of DOACs than standard-doses of DOACs in patients with a higher body mass index (BMI) of approximately 31 kg/m^2^, whereas similar stroke/SE and death rates (Briasoulis et al., 2020) were observed between the two groups of patients with a lower BMI of approximately 24 kg/m^2^. Similarly, the standard dose of rivaroxaban in Japan is 15 mg instead of 20 mg in other countries based on pharmacokinetic data in Japanese adults (Murata et al., 2019), who have a relatively lower BMI than Europeans and Americans. Nevertheless, the clinical outcomes were not significantly different between under-dosing and on-label dosing in different geographic regions (Asia and North America). Accordingly, reduced doses of DOACs might be safely and effectively used in clinical practice, especially for Asian patients.

Over-dosing of DOACs was not as frequent as under-dosing, with a rate of 3.4–8.4% (Steinberg et al., 2016; Murata et al., 2019; Yu et al., 2020). According to the results of this study, a significant association was detected between over-dosing of DOACs and increased risk of stroke/SE, major bleeding, and death, compared with standard dosing. These results are in accordance with those of the ORBIT-AF II study, in which higher all-cause mortality was observed with over-dosing of DOACs (Steinberg et al., 2016). Clinicians might tailor the dose of DOACs according to the underlying risks, regardless of the label recommendation and the favourable risk-benefit profiles across risk strata demonstrated by large clinical trials (Steinberg et al., 2016). In this study, over-dosing of DOACs was associated with significantly increased risks of stroke/SE, major bleeding, and death compared with on-label dosing of DOACs in Asian patients, but there was no significant difference in patients in other regions. The results were in accordance with the results in a Korean database study (Steinberg et al., 2016; Murata et al., 2019; Yu et al., 2020) and a Japanese SAKURA AF study (Okumura et al., 2017; Lee et al., 2019), suggesting that Asian patients might be more intolerant to high-dose DOACs. Nevertheless, the results were difficult to explain, as it is generally accepted that a higher dose of anticoagulants would be associated with a decreased risk of stroke/SE. Therefore, more studies should be conducted to look into this problem, and patients allocated to an over-dose of DOAC should be carefully and intensively followed up.

### Study Strengths and Limitations

The strength of this study is that we applied a systematic and rigorous approach to evaluate the real-world benefits and negative effects of non-standard dosing of DOACs in patients with AF. We estimated the quality of the included studies using the revised NOS tool, performed subgroup analyses according to individual DOACs and different regions, and conducted sensitivity analyses to strengthen the robustness of the results. Nevertheless, the study had some limitations. First, because of the observational nature of the studies, unavoidable selection bias limits the generalisation and extrapolation of the results to clinical practice. Future large-scale trials are required to validate the outcomes of non-standard dosing of DOACs. However, it could not be neglected that deliberate under- or over-dosing might not be feasible in clinical practice. Second, due to the limited number of inclusive studies, the results should be considered with caution. However, our results could be credible considering the low degree of heterogeneity in both overall and subgroup analyses.

## Conclusion

Similar clinical outcomes were observed between under-dosing and on-label dosing of DOACs in patients with AF. A significant association was observed between over-dosing of DOACs and increased risks of stroke/SE, major bleeding, and death compared with on-label dosing. Overall, a reduced dose of DOACs might be safely and effectively used in clinical practice, especially for Asian patients, whereas high-dose DOACs may not be well tolerated by Asian patients.

## Data Availability

The original contributions presented in the study are included in the article/Supplementary Material, further inquiries can be directed to the corresponding authors.
