# Supplementary material for: Effectiveness and Safety of Under or Over-dosing of Direct Oral Anticoagulants in Atrial Fibrillation: A Systematic Review and Meta-analysis of 148909 Patients From 10 Real-World Studies
Source: Front Pharmacol. 2021 Mar 18;12:645479. doi: 10.3389/fphar.2021.645479 (PMC8012667; doi:10.3389/fphar.2021.645479)
Supplement: Supplementary file 1 [file datasheet1.pdf]

SUPPLEMENTAL FILE

Title: Effectiveness and safety of off-label doses of direct oral anticoagulants in atrial fibrillation: Insights from the pooled-analysis of 148909 patients from 10 real-world studies

Contents

eTable 1. Search strategy to identify studies reporting DOACs under- or over-doses

**Literature databases**	**Search items**	**Items found**
MEDLINE via PUBMED	#1<br>“dabigatran”[MeSH Terms] OR “dabigatran”[Title/Abstract] OR “Pradaxa”[Title/Abstract] OR “rivaroxaban”[MeSH Terms] OR “rivaroxaban”[Title/Abstract] OR “Xarelto”[Title/Abstract] OR “apixaban” [MeSH Terms] OR “apixaban”[Title/Abstract] OR “Eliquis”[Title/Abstract] OR “edoxaban”[MeSH Terms] OR “edoxaban”[Title/Abstract] OR “Savaysa”[Title/Abstract]) OR “betrixaban”[MeSH Terms] OR “betrixaban”[Title/Abstract] OR “Bevyxxa”[Title/Abstract]) OR “Non-vitamin K antagonist oral anticoagulants”[Title/Abstract] OR “NOACs”[Title/Abstract]) OR “direct oral anticoagulants”[Title/Abstract]) OR “DOACs”[Title/Abstract]) OR “novel oral anticoagulants”[Title/Abstract]) OR “new oral anticoagulants”[Title/Abstract]) OR “factor Xa inhibitors”[Title/Abstract]) OR “factor IIa inhibitors”[Title/Abstract]	1017

	#2<br>“label”[Title/Abstract] OR “off-label” [Title/Abstract] OR “on-label” [Title/Abstract] OR “underdosing” [Title/Abstract] OR “underdose”[Title/Abstract] OR “underdosed”[Title/Abstract] OR “overdosing”[Title/Abstract] OR “overdose”[Title/Abstract] OR “overdosed”[Title/Abstract] OR “reduced dose” [Title/Abstract] OR “low dose”[Title/Abstract] OR “prescribing patterns”[Title/Abstract] OR “dosing patterns”[Title/Abstract]<br>#1 AND #2	
EMBASE	#1<br>‘dabigatran’/exp OR ‘dabigatran’:ti,ab,kw OR ‘Pradaxa’: ti,ab,kw OR ‘rivaroxaban’/exp OR ‘rivaroxaban’: ti,ab,kw OR ‘Xarelto’: ti,ab,kw OR ‘apixaban’/exp OR ‘apixaban’: ti,ab,kw OR ‘Eliquis’: ti,ab,kw OR edoxaban’/exp OR ‘edoxaban’: ti,ab,kw OR ‘Savaysa’: ti,ab,kw OR ‘betrixaban’/exp OR ‘betrixaban’: ti,ab,kw OR ‘Bevyxxa’: ti,ab,kw OR ‘Non-vitamin K antagonist oral anticoagulants’: ti,ab,kw OR ‘NOACs’: ti,ab,kw OR ‘direct oral anticoagulants’: ti,ab,kw OR ‘DOACs’: ti,ab,kw OR ‘novel oral anticoagulants’: ti,ab,kw OR ‘new oral anticoagulants’: ti,ab,kw OR ‘factor Xa inhibitors’: ti,ab,kw OR ‘factor IIa inhibitors’: ti,ab,kw<br>#2<br>‘label’:ti,ab,kw OR ‘off-label’:ti,ab,kw OR ‘on-label’:ti,ab,kw OR‘underdosing’:ti,ab,kw OR ‘underdose’:ti,ab,kw OR ‘underdosed’:ti,ab,kw OR ‘overdosing’:ti,ab,kw OR ‘overdose’:ti,ab,kw OR ‘overdosed’:ti,ab,kw OR‘reduced dose’:ti,ab,kw OR ‘low dose’:ti,ab,kw OR ‘prescribing patterns’:ti,ab,kw OR ‘dosing patterns’:ti,ab,kw<br>#1 AND #2	1127
COCHRANE	#1	193

	MeSH descriptor: [dabigatran] OR dabigatran: ti,ab,kw OR Pradaxa: ti,ab,kw ORMeSH descriptor: [rivaroxaban] OR rivaroxaban: ti,ab,kw OR Xarelto: ti,ab,kw OR MeSH descriptor: [apixaban] OR apixaban: ti,ab,kw OR Eliquis: ti,ab,kw OR MeSH descriptor: [edoxaban] OR edoxaban: ti,ab,kw OR Savaysa: ti,ab,kw OR MeSH descriptor: [betrixaban] OR betrixaban: ti,ab,kw OR Bevyxxa: ti,ab,kw OR Non-vitamin K antagonist oral anticoagulants: ti,ab,kw ORNOACs: ti,ab,kw OR direct oral anticoagulants: ti,ab,kw OR DOACs: ti,ab,kw OR novel oral anticoagulants: ti,ab,kw OR new oral anticoagulants: ti,ab,kw OR factor Xa inhibitors: ti,ab,kw OR factor IIa inhibitors: ti,ab,kw<br>#2<br>label:ti,ab,kw OR off-label:ti,ab,kw OR on-label:ti,ab,kw OR underdosing:ti,ab,kw OR underdose:ti,ab,kw OR underdosed:ti,ab,kw OR overdosing:ti,ab,kw OR overdose:ti,ab,kw OR overdosed:ti,ab,kw OR reduced dose:ti,ab,kw OR low dose:ti,ab,kw OR prescribing patterns:ti,ab,kw OR dosingpatterns:ti,ab,kw<br>#1 AND #2	
Overall		2337
Duplication		279

eTable 2. Quality assessment scale

Bias type	Selection (sample population)	Selection (sample size)	Selection (participation rate)	Performance bias (outcome assessment)	Performance bias(analytical methods to control for bias)
**Low risk (score=2)**	1) Sample from the general population, not a select group;<br>2) Consecutive unselected population;<br>3) Rationale for case and control selection explained.	1) Sample size calculation performed and adequate.	1) High response rate (>85%).	1) Diagnosis using consistent criteria and direct examination.	1) Analysis appropriate for the type of sample (subgroup analysis/regression etc.)
**Moderate risk (score=1)**	1) Sample selected from large population but selection criteria not defined;<br>2) Sample selection ambiguous but may be representative;<br>3) Rationale for cases and controls not explained;	1) Sample size calculation performed and reasons for not meeting sample size given;<br>2) Sample size calculation not performed but all	1) Moderate response rate (70-85%).	1) Assessment from administrative database or register;<br>2) Assessment from hospital record or interviewer.	1) Analysis does not account for common adjustment.

	4) Eligibility criteria not explained;<br>5) Analysis to adjust for sampling strategy bias.	eligible persons studied.			
**High risk (score=0)**	1) Highly select population making it difficult to generalize finding;<br>2) Sample selection ambiguous and sample unlikely to be representative.	1) Sample size estimation unclear or only sub-sample studied.	1) Low response rate (<70%);<br>2) Response rate not reported.	1) Assessment from non-validated data or generic estimate from the overall population.	1) Data confusing.

eTable 3. Excluded studies with reasons

Excluded Studies	Drugs	Reason for exclusion
McAliste,2018(McAlister et al., 2018)	Direct oral anticoagulants	No outcome data
Leef, 2019(Leef et al., 2019)	Direct oral anticoagulants	No outcome data
Lee, 2019-A(Lee and Lee, 2019)	Direct oral anticoagulants	No outcome data
Garcia Rodrigue, 2019(Garcia Rodriguez et al., 2019)	Direct oral anticoagulants	No outcome data
Falissard, 2019(Falissard et al., 2019)	Apixaban	No outcome data
Draper, 2017(Draper et al., 2017)	Direct oral anticoagulants	No outcome data
De Caterina, 2019(De Caterina et al., 2019)	Edoxaban	No outcome data
Bell, 2016(Bell et al., 2016)	Direct oral anticoagulants	No outcome data
Jacobs, 2019(Jacobs et al., 2019)	Direct oral anticoagulants	No outcome data
Okumura, 2017(Okumura et al., 2017)	Direct oral anticoagulants	No outcome data
Basaran, 2016(Basaran et al., 2016)	Direct oral anticoagulants	No outcome data
Lee, 2019-B(Lee et al., 2019)	Direct oral anticoagulants	The same data source
Lee, 2020(Lee and Choi, 2020)	Direct oral anticoagulants	No off-label DOACs dosing data
Soo Cho, 2020(Kakkar et al., 2013)	Direct oral anticoagulants	The same data source
Inoue, 2019(Inoue et al., 2020)	Apixaban	No adjusted data
Navarro-Almenzar, 2019(Navarro-Almenzar et al., 2019)	Direct oral anticoagulants	No adjusted data
Yamaji, 2017(Yamaji et al., 2017)	Direct oral anticoagulants	No adjusted data
Yiginer 2017(Yiginer et al., 2017)	Rivaroxaban	No outcome data

Viprey 2016(Viprey et al., 2016)	Direct oral anticoagulants	No outcome data
Vinter 2019(Vinter et al., 2019)	Apixaban	No off-label dosing data
Umei 2017(Umei et al., 2017)	Direct oral anticoagulants	No outcome data
Tellor 2017(Tellor et al., 2017)	Apixaban	No outcome data
Tellor 2015(Tellor et al., 2015)	Rivaroxaban	No outcome data
Suwa 2019(Suwa et al., 2019)	Apixaban	No outcome data
Sieg 2015(Sieg and Nappi, 2015)	Direct oral anticoagulants	No outcome data
Shrestha 2018(Shrestha et al., 2018)	Direct oral anticoagulants	No outcome data
Schwartz 2017(Schwartz et al., 2017)	Direct oral anticoagulants	No outcome data
Saunders 2019(Saunders et al., 2019)	Direct oral anticoagulants	Not AF patients data
Sato 2018(Sato et al., 2018)	Direct oral anticoagulants	No outcome data
Pisters 2017(Pisters et al., 2017)	Direct oral anticoagulants	No outcome data
Paciaroni 2019(Paciaroni et al., 2019)	Direct oral anticoagulants	Case control study
Moudallel 2018(Moudallel et al., 2018)	Direct oral anticoagulants	No outcome data
Lavoie 2016(Lavoie et al., 2016)	Direct oral anticoagulants	No outcome data
Kim 2019(Kim et al., 2019)	Direct oral anticoagulants	Not off-label dose study
Khan 2016(Khan et al., 2016)	Direct oral anticoagulants	No outcome data
Kato 2018(Kato et al., 2018)	Direct oral anticoagulants	No outcome data
Kartas 2019(Kartas et al., 2019)	Direct oral anticoagulants	No outcome data
Jones 2020(Jones et al., 2020)	Rivaroxaban	No outcome data

Hussain 2013(Hussain et al., 2013)	Dabigatran	No outcome data
Howerton 2019(Howerton et al., 2019)	Direct oral anticoagulants	No outcome data
Hirsh Raccah 2019(Hirsh Raccah et al., 2019)	Direct oral anticoagulants	No outcome data
Gibson 2018(Gibson et al., 2018)	Apixaban	No outcome data
Galaune 2019(Galaune et al., 2019)	Direct oral anticoagulants	No outcome data
Eschler 2019(Eschler et al., 2019)	Direct oral anticoagulants	Not AF patients data
Chowdhry 2016(Chowdhry et al., 2016)	Dabigatran	No outcome data
Chen 2018(Chen and Lin, 2018)	Direct oral anticoagulants	No outcome data
Chan 2018(Chan et al., 2018)	Apixaban	Not off-label dose study
CainzosAchirica 2018(Cainzos-Achirica et al., 2018)	Dabigatran	No off-label dosing data
Buchholz 2018(Buchholz et al., 2018)	Apixaban	No outcome data
Bruneau 2019(Bruneau et al., 2019)	Direct oral anticoagulants	No outcome data
Barra 2016(Barra et al., 2016)	Direct oral anticoagulants	No outcome data
Alali 2019(Alali et al., 2019)	Dabigatran	No outcome data
Ablefoni 2019(Ablefoni and Buchholz, 2019)	Rivaroxaban	No outcome data

eTable 4. Detailed definition of DOACs under- or over-doses in the included studies

Study	Off-label definition
Benjamin A, 2016	Underdosed and overdosed DOACs were categorized according to U.S. FDA-approved package inserts (PIs). Dabigatran: 150 mg twice daily is standard dose; CrCl 30 to 50 mL/min: No dosage adjustment necessary unless patient receiving concomitant dronedarone, then consider reducing dabigatran to 75mg twice daily; CrCl 15 to 30 mL/min: 75 mg twice daily unless patient receiving concomitant dronedarone, then avoid concurrent use; CrCl <15 mL/min or on dialysis: not recommended. Rivaroxaban: 20 mg once daily is standard dose; CrCl 15 to 50 mL/min: 15 mg once daily; CrCl <15mL/min or on dialysis: Avoid use. Apixaban: 5 mg twice daily unless patient has any 2 of the following: Age ≥80 years,<br>body weight ≤60 kg, or serum creatinine ≥1.5 mg/dL, then reduce dose to 2.5mg twice daily. On dialysis: 5 mg twice daily; reduce to 2.5 mg twice daily if age ≥80 years or body weight ≤60 kg.
Cheng, 2019	Underdosed and overdosed DOACs were categorized according to ROCKET-AF or J-ROCKET dosage criteria. on-label dosing: patients received daily dose of rivaroxaban according to ROCKET-AF (20mg/d for patients with an eGFR ≥50 mL/min and 15 mg/d for those with an eGFR=30–49 mL/min) or J-ROCKET (15 mg/d for patients with an eGFR ≥50 mL/min and 10 mg/d for those with an eGFR=30–49 mL/min); off-label low-dosing: rivaroxaban at a daily dose of 10mg for patients with an eGFR ≥50 mL/min.
Yao, 2017	Underdosed and overdosed DOACs were categorized according to U.S. FDA-approved package inserts (PIs).Patients with a renal indication for dose reduction but who received standard dose DOACs (potential overdosing), patients with no renal indication but receiving reduced dose DOACs (potential underdosing).Patients were considered to have a renal indication for dose reduction if they were prescribed dabigatran and had an eGFR <30 ml/min/1.73 m^2, rivaroxaban and an eGFR

	<50 ml/min/1.73 m^2, The indication for dose reduction with apixaban requires 2 of the following 3 criteria: age≥80 years, weight<60 kg, and SCr level ≥1.5 mg/dl.
Murata, 2018	Underdosed and overdosed DOACs were categorized according to Japan-approved package inserts (PIs).The following low-dose regimens were considered to be appropriate: dabigatran, 110 mg (b.i.d.), for patients with a CrCl of 30–50 mL/min, age ≥70 years and a prior history of bleeding; rivaroxaban, 10 mg (o.d.), for patients with a CrCl of 15–50 mL/min; apixaban, 2.5 mg(b.i.d.), for patients with any 2 of the following characteristics: ≥80 years, body weight <60 kg and serum Cr level≥1.5 mg/dL; and edoxaban, 30 mg (o.d.), for patients with a CrCl of 15–50 mL/min or body weight is <60 kg.
Arbel, 2019	Off-label dose-Reduced DOACs were in compliance with the approved label of Israel.
Ikeda, 2019	10 mg rivaroxaban in Japanese AF patients with CrCl ≥ 50 mL/min is considered as under-dose.
Briasoulis, 2020	DOAC prescriptions do not adhere to the Food and Drug Administration (FDA) dosing criteria. Dabigatran: 150 mg twice daily is standard dose. CrCl 30 to 50 mL/min: No dosage adjustment necessary unless patient; receiving concomitant dronedarone, then consider reducing dabigatran to 75mg twice daily; CrCl 15 to 30 mL/min: 75 mg twice daily unless patient receiving concomitant dronedarone, then avoid concurrent use. Rivaroxaban: 20 mg once daily is standard dose. CrCl 15 to 50 mL/min: 15 mg once daily.
Salameh, 2020	The recommended standard dose of apixaban is 5 mg bid. The use of apixaban at reduced adjusted dose (2.5 mg bid) if at least one of the following two criteria is fulfilled: eGFR of 15-29 ml/min or at least two of the following: age ≥80 years, weight ≤60kg or serum creatinine ≥1.5 mg/dL.
Yu, 2020	Dose reduction criteria is based on Korean label. Dabigatran: 150 mg twice daily is standard dose; CrCl 30 to 50 mL/min: 110 mg twice daily, if any of the following: CrCl30-50 mL/min, age ≥75 years. Rivaroxaban: 20 mg once daily is standard

	dose;15mg once daily if CrCl 15-49mL/min. Apixaban: 5 mg twice daily unless patient has any 2 of the following: 2.5 mg twice daily, if at least 2of age ≥80 years, body weight ≤60kg or serum creatinine level≥1.5 mg/dL 2.5 mg twice daily, if CrCl 15-29 mL/min. Edoxaban: 60 mg once daily is standard dose; 30 mg once daily if any of the following: CrCl of15-50mL/min, body weight ≤60kg, concomitant use of glycoprotein in inhibitors.
Almeida, 2020	The standard dose is 5 mg/bid for apixaban, 20 mg/qd for rivaroxaban, and 150 mg/qd for dabigatran; the underdosed is 2.5 mg bid. For apixaban, 15 mg qd for rivaroxaban and 75 mg/qd for dabigatran.

DOACs: direct oral anticoagulants; eGFR: estimated glomerular filtration rate; CrCl: creatinine clearance.

eTable 5. Detailed definition of clinical outcomes in the included studies

**Study**	**Clinical outcomes definition**
Benjamin A, 2016	The outcomes of interest included all-cause death, stroke or systemic embolism, myocardial infarction, and major bleeding classified by International Society on Thrombosis and Haemostasis criteria.
Cheng, 2019	The clinical end points included ischemic stroke, intracranial hemorrhage. Patients were followed up until the occurrence of clinical end points or their last visits, which could be traced within the electrical medical record.
Yao, 2017	The stroke and major bleeding outcomes were identified using ICD-9 codes in the primary or secondary diagnosis positions of inpatient claims. Stroke or systemic embolism included ischemic stroke (ICD-9-CM codes: 433.x1, 434.x1, or 436), and systemic embolism (ICD-9-CM codes: 444.x). Major bleeding was categorized according to the International Society on Thrombosis and Haemostasis (ISTH) criteria: (1) Fatal bleeding, and/or, (2) Symptomatic bleeding in a critical area or organ, such as intracranial, intraspinal, intraocular, retroperitoneal, intraarticular or pericardial, or intramuscular with compartment syndrome, and/or, (3) Bleeding causing a fall in hemoglobin level of 2 g/dL or more, or leading to transfusion of two or more units of whole blood or red cells.
Murata, 2018	Major bleeding was defined as a reduction in the Hb level of at least 2 g/dL, transfusion of at least 2 units of blood or symptomatic bleeding in a critical area or organ, and was specified as the endpoint of safety.
Arbel, 2019	The clinical outcomes were based on the primary ICD-9- CM Codes. Ischemic stroke: 433.x1, 434.x1; myocardial infarction: 410.x; intracranial hemorrhage: 430, 431, 432.x; gastrointestinal bleeding: 456.0, 456.20, 530.21, 530.7, 530.82, 531.0x, 531.2x, 531.4x, 531.6x, 532.0x, 532.2x, 532.4x, 532.6x, 533.0x, 533.2x, 533.4x, 533.6x, 534.0x, 534.2x, 534.4x, 534.6x, 535.x1, 537.83, 537.84, 562.02, 562.03, 562.12, 562.13 568.81, 569.3, 569.85, 578.x.
Ikeda, 2019	The outcomes were major bleeding, stroke, and myocardial infarction. Major bleeding was defined as the International Society of Thrombosis and Haemostasis (ISTH) criteria. Myocardial infarction is defined as typical symptoms plus

	elevation in the levels of cardiac biomarkers (troponin I, troponin T, or creatinine kinase-MB) above the upper limit of normal, new pathological Q waves in ≥ 2 contiguous electrocardiographic leads, or confirmation at autopsy.
Briasoulis, 2020	The clinical outcomes were based on the primary ICD-9- CM diagnosis. Ischemic stroke: 433*, 434*, 436*, 4371, 4378, 4379. Gastrointestinal bleeding: 4552, 4555, 4558, 4560, 45620, 5307, 53082, 5310-5316, 5320-5326, 5330-5336, 5340-5346, 53501-53561, 56202-56203, 56212-56213, 5693, 56985, 5780, 5781, 5789, 53783, 56881. Major bleeding: 59970, 59971, 59972, 71911, 7847*, 7848x, 7863*, 4230*, 4590*, 852*, 853*.
Salameh, 2020	The efficacy outcomes were defined as hospital discharge diagnoses of ischemic stroke (ICD 9 codes: 433.x1, 434.x1, 436) or systemic arterial embolism (ICD 9 codes: 444.x, 445.x). Safety outcomes were defined as a hospital discharge diagnosis of major bleeding including intracranial hemorrhage and major gastrointestinal bleeding.
Yu, 2020	The outcomes were stroke or systemic embolism, major bleeding, death, intracranial bleeding, gastrointestinal bleeding, and myocardial infarction. Stroke or systemic embolism was defined from diagnosis of ischemic stroke with concomitant imaging studies of the brain or related death. Intracranial hemorrhage (ICH) was defined from admission diagnosis of ICH with concomitant imaging studies of the brain or related death. Gastrointestinal bleeding was defined from admission diagnosis or related death. Major bleeding was defined from ICH, gastrointestinal bleeding, or anemia caused by bleeding. Myocardial infarction was defined from admission diagnosis of MI with concomitant use of dual antiplatelet therapy or related death.
Almeida, 2020	The outcome included stroke or systemic embolism, and major bleeding.

DOACs: direct oral anticoagulants; eGFR: estimated glomerular filtration rate; CrCl: creatinine clearance; MI: myocardial infarction.

eTable 6. Detailed demographics and clinical characteristics of the included studies

Study	Mean age (y)	Female (%)	HF (%)	HBP (%)	DM (%)	TIA (%)	MI (%)	Co-antiplatelet agents	BMI (kg/m^2)	CrCl (mL/min)	CHA2DS2-VASc>2	CHADS2-VASc (mean)	HAS-BLED(mean)	Vascular disease
Benjamin A, 2016(Steinberg et al., 2016)	71.0	41.8	20.7	NR	NR	NR	NR	NR	31.4	89.2	87.0	NR	NR	NR
Cheng, 2019(Cheng et al., 2019)	75.7	36.0	25.5	56.9	21.9	2.30	NR	23.7	NR	NR	NR	2.9	NR	NR
Yao, 2017(Yao et al., 2017)	77.5	49.5	51.7	97.8	54.2	NR	NR	12.2	NR	NR	99.5	NR	NR	43.2
Murata, 2018(Murata et al., 2019)	71.7	28.5	NR	69.4	21.6	10.1	NR	12.7	24.1	70.5	NR	2.9	1.3	11.7
Arbel, 2019(Morris et al., 2019)	76.0	52.5	28.5	96.0	59.0	NR	NR	43.5	30.0	NR	NR	4.7	NR	17.5
Ikeda, 2019(Ikeda et al., 2019)	71.2	33.5	22.1	74.9	23.3	21.2	NR	13.5	24.6	75.5	NR	3.2	1.4	3.4

Briasoulis, 2020(Briasoulis et al., 2020)	51.2	52.3	NR	NR	NR	NR	NR	NR	NR	NR	NR	NR	NR	NR
Salameh, 2020(Kakkar et al., 2013)	78.7	51.7	32.6	90.1	46.4	10.3	31.9	48.9	NR	63.8	NR	4.8	2.8	NR
Yu, 2020(Connolly et al., 2009)	70.5	39.7	61.2	96.3	31.6	45.5	11.7	25.5	NR	NR	NR	4.6	NR	28.8
Almeida, 2020(Patel et al., 2011)	84.0	65.9	70.5	85.5	36.4	23.0	NR	NR	NR	75.8	NR	5.1	2.5	15.8

BMI: Body Mass Index; CrCl: creatinine clearance rate; DM: Diabetes; HF: heart failure; HBP: hypertension; TIA: transient ischemic attack;MI: myocardial infarction; NR: not reported.

eTable 7. Quality scores of the included studies

Study	Sample population	Sample size	Participation rate	Outcome assessment	Analytical methods to control for bias	Total score
Benjamin A, 2016	2	2	2	1	2	9
Cheng, 2019	2	2	2	1	2	9
Yao, 2017	2	2	2	1	2	9
Murata, 2018	2	2	2	1	2	9
Arbel, 2019	2	2	2	1	2	9
Ikeda, 2019	2	2	2	1	2	9
Briasoulis, 2020	2	2	2	1	2	9
Salameh, 2020	2	2	2	1	2	9
Yu, 2020	2	2	2	1	2	9
Almeida, 2020	2	2	2	1	1	8

eTable 8. Sensitivity analysis of clinical outcome in under-dose DOACs

Study omitted	Stroke: HR (95%Cl)	Major bleeding : HR (95%Cl)	ICH: HR (95%Cl)	GIB: HR (95%Cl)	Death : HR (95%Cl)	MI: HR (95%Cl)
Benjamin A, 2016	1.01 (0.93-1.09)	1.01 (0.77-1.24)			1.41 (0.95-1.87)	1.07 (0.88-1.25)
Cheng, 2019	1.00 (0.92-1.09)					
Murata, 2018	1.01 (0.93-1.09)	1.05 (0.84-1.25)			1.35 (0.98-1.72)	
Arbel, 2019	1.01 (0.92-1.09)				1.10 (0.98-1.21)	1.10 (0.90-1.29)
Ikeda, 2019	1.01 (0.92-0.09)	1.01 0.76-1.26)	1.04 (0.61-1.47)			1.07 (0.89-1.25)
Briasoulis, 2020	1.01 (0.93-1.10)	0.94 (0.68-1.21)	1.11 (0.73-1.49)	1.19 (0.57-1.81)		
Salameh, 2020	1.01 (0.92-1.09)	0.90 (0.74-1.07)	0.99 (0.67-1.33)	0.96 (0.81-1.12)		
Yu, 2020	1.04 (0.87-1.20)	0.96 (0.66-1.27)	0.99 (0.56-1.43)	1.27 (0.82-1.73)	1.54 (1.28-1.79)	0.98 (0.54-1.42)
Cheng, 2019			1.22 (0.97-1.47)			
Almeida, 2020					1.37 (0.94-1.79)	

CI: confidence interval.

eTable 9. Sensitivity analysis of clinical outcome in over-dose DOACs

**Study omitted**	**Stroke:** **HR (95%Cl)**	**Major bleeding :** **HR (95%Cl)**	**ICH:** **HR (95%Cl)**	**GIB:** **HR (95%Cl)**	**Death :** **HR (95%Cl)**	**MI:** **HR (95%Cl)**
Benjamin A, 2016	1.18 (1.04-1.32)	1.15 (1.02-1.29)			1.19 (1.01-1.37)	1.01 (0.73-1.29)
Murata, 2018	1.18 (1.04-1.32)	1.16 (1.03-1.29)			1.26 (0.85-1.66)	
Briasoulis, 2020	1.17 (1.00-1.33)	1.21 (1.03-1.39)	1.27 (0.80-1.74)	1.17 (0.96-1.38)		
Yu, 2020	1.24 (0.95-1.52)	1.20 (0.89-1.51)	1.16 (0.78-1.53)	1.08 (0.97-1.28)	1.95 (0.75-3.15)	0.31 (0.00-1.26)
Yao, 2017	1.18 (1.04-1.32)	1.16 (1.02-1.29)				

CI: confidence interval.

eTable 10. Meta-regression of clinical outcome in under-dose DOACs

Variables	Stroke*: P* value	MI*: P* value	Death*: P* value	Major bleeding*:* *P* value	ICH*: P* value	GIB*: P* value
Mean age	0.800	0.642	0.657	0.773	0.666	0.813
Female	0.907	0.817	0.949	0.734	0.877	0.831
HF	0.135	0.290	0.469	0.524	0.335	
HBP	0.216	0.532	0.375	0.425	0.471	
DM	0.862	0.938	0.724	0.682	0.696	
TIA	0.194		0.422	0.333	0.276	
MI				0.563		
Co-antiplatelet agents	0.657	0.978	0.793	0.563	0.649	
BMI	0.679	0.704	0.687	0.951		
CrCl	0.646		0.681	0.783		
CHADS2-VASc	0.261		0.703	0.470	0.526	
HAS-BLED	0.469			0.533		
Vascular disease	0.265	0.443	0.400	0.458		

BMI: Body Mass Index; CrCl: creatinine clearance rate; DM: Diabetes; HF: heart failure; HBP: hypertension; TIA: transient ischemic attack; MI: myocardial infarction

eTable 11. Meta-regression of clinical outcome in over-dose DOACs

**Variables**	**Stroke*: P* value**	**Major bleeding*: P* value**	**Death*: P* value**	**ICH*: P* value**	**GIB*: P* value**	**MI*: P* value**
Mean age	0.861	0.961	0.813			
Female	0.902	0.721	0.831			
HF	0.464	0.486				
HBP	0.550	0.566				
DM	0.924	0.863				
TIA						
MI	0.413					
Co-antiplatelet agents		0.432				
BMI						
CrCl						
CHADS2-VASc	0.796					
HAS-BLED	0.861					
Vascular disease	0.902	0.851				

BMI: Body Mass Index; CrCl: creatinine clearance rate; DM: Diabetes; HF: heart failure; HBP: hypertension; TIA: transient ischemic attack; MI: myocardial infarction

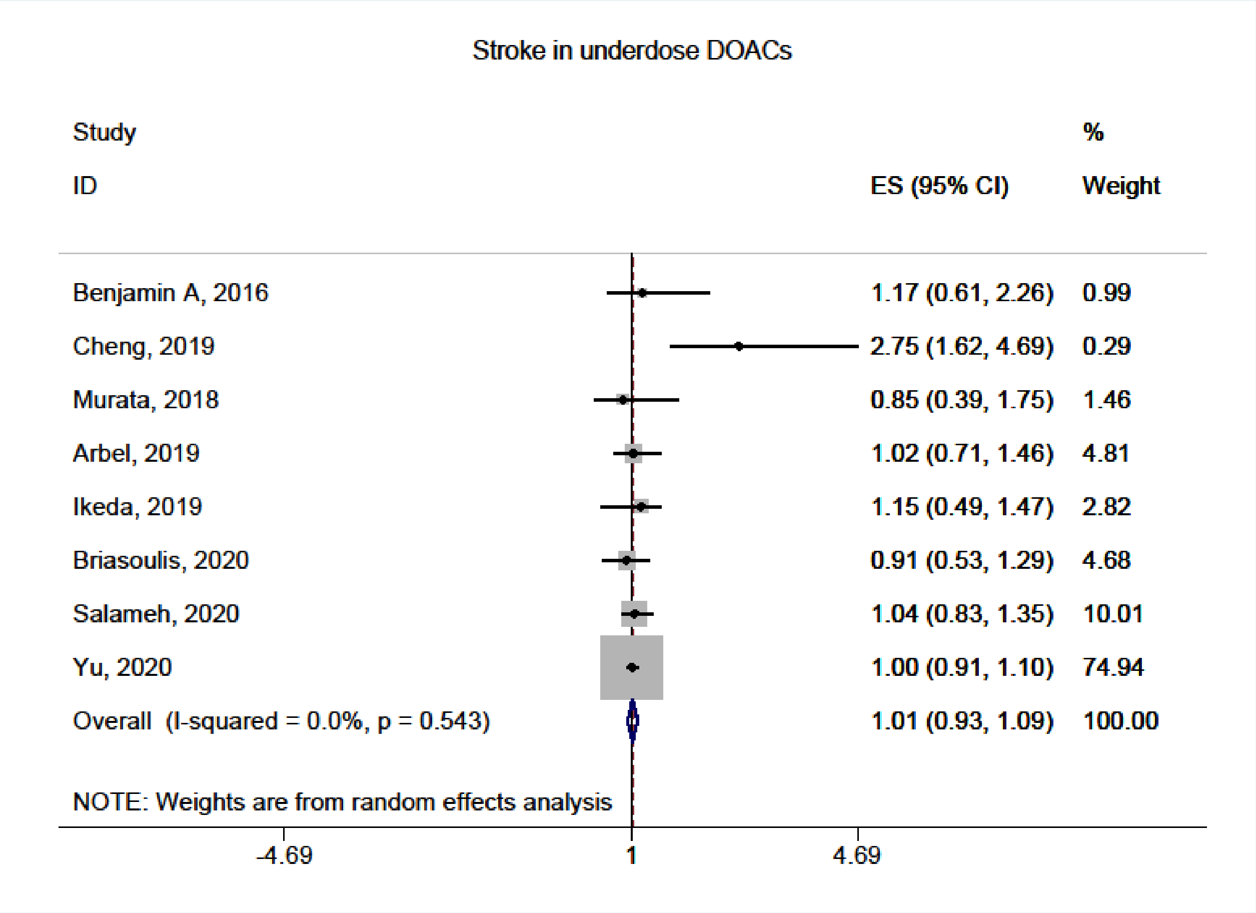

eFigure 1. Pooled stroke in under-dosing of DOACs

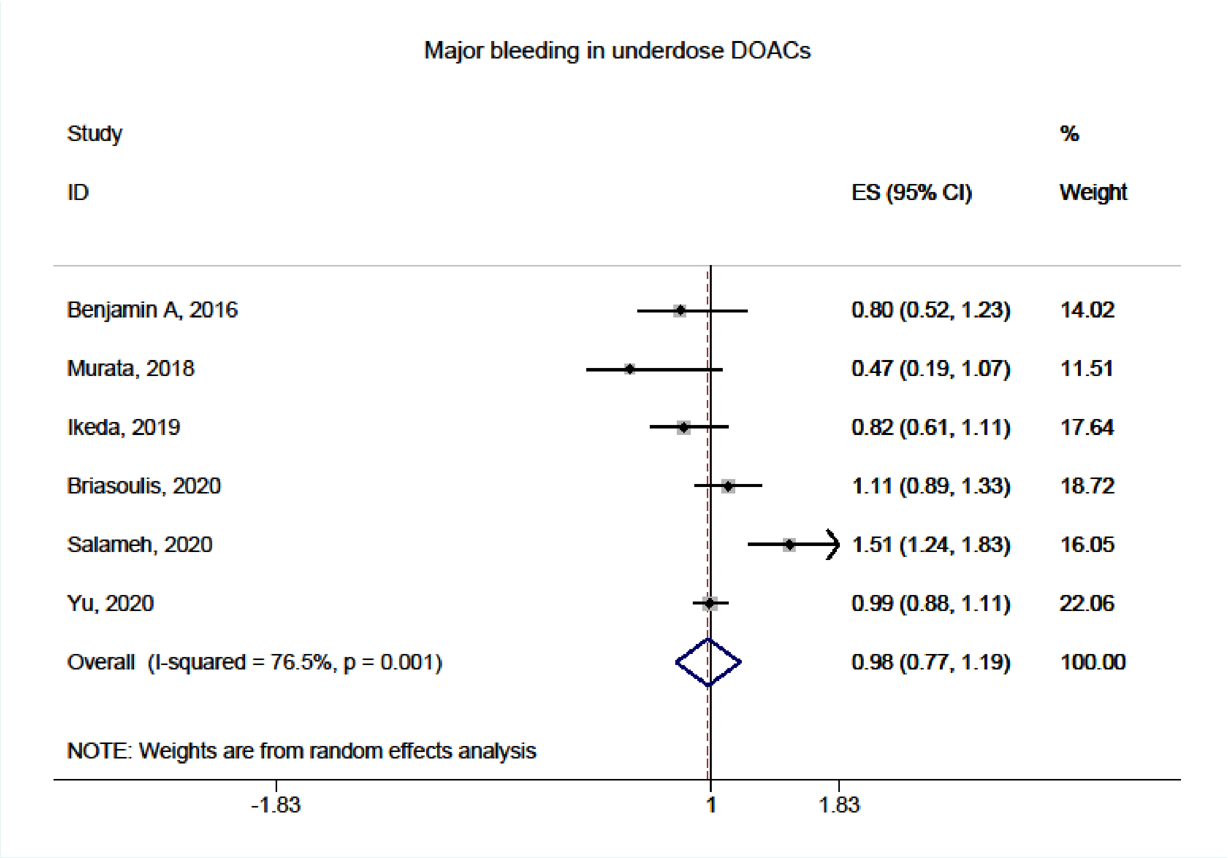

eFigure 2. Pooled major bleeding in under-dosing of DOACs

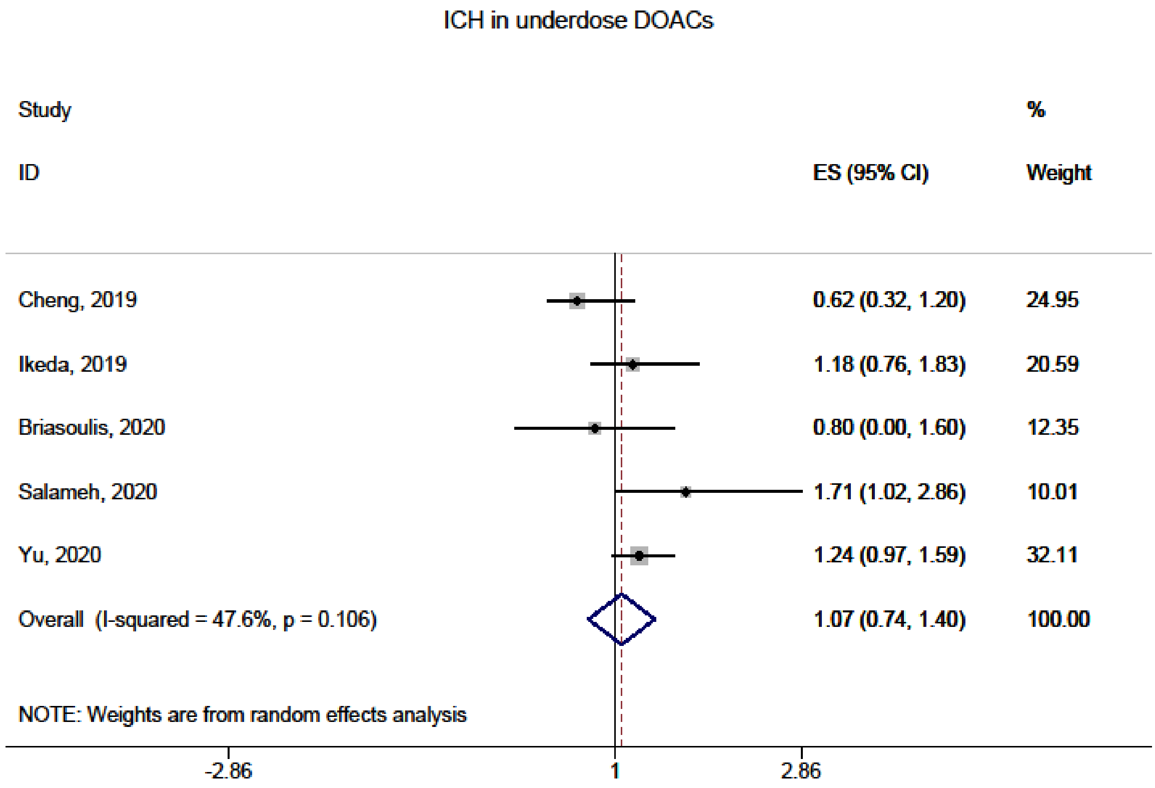

eFigure 3. Pooled ICH in under-dosing of DOACs

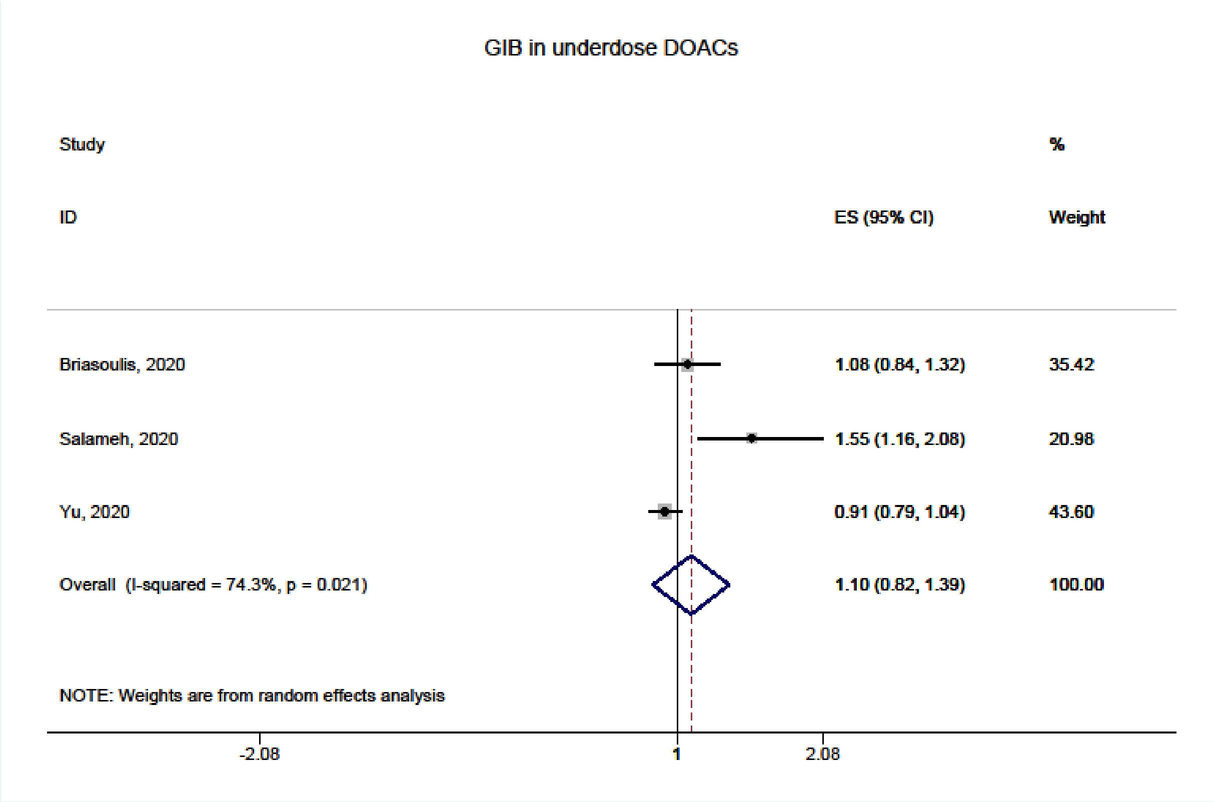

eFigure 4. Pooled GIB in under-dosing of DOACs

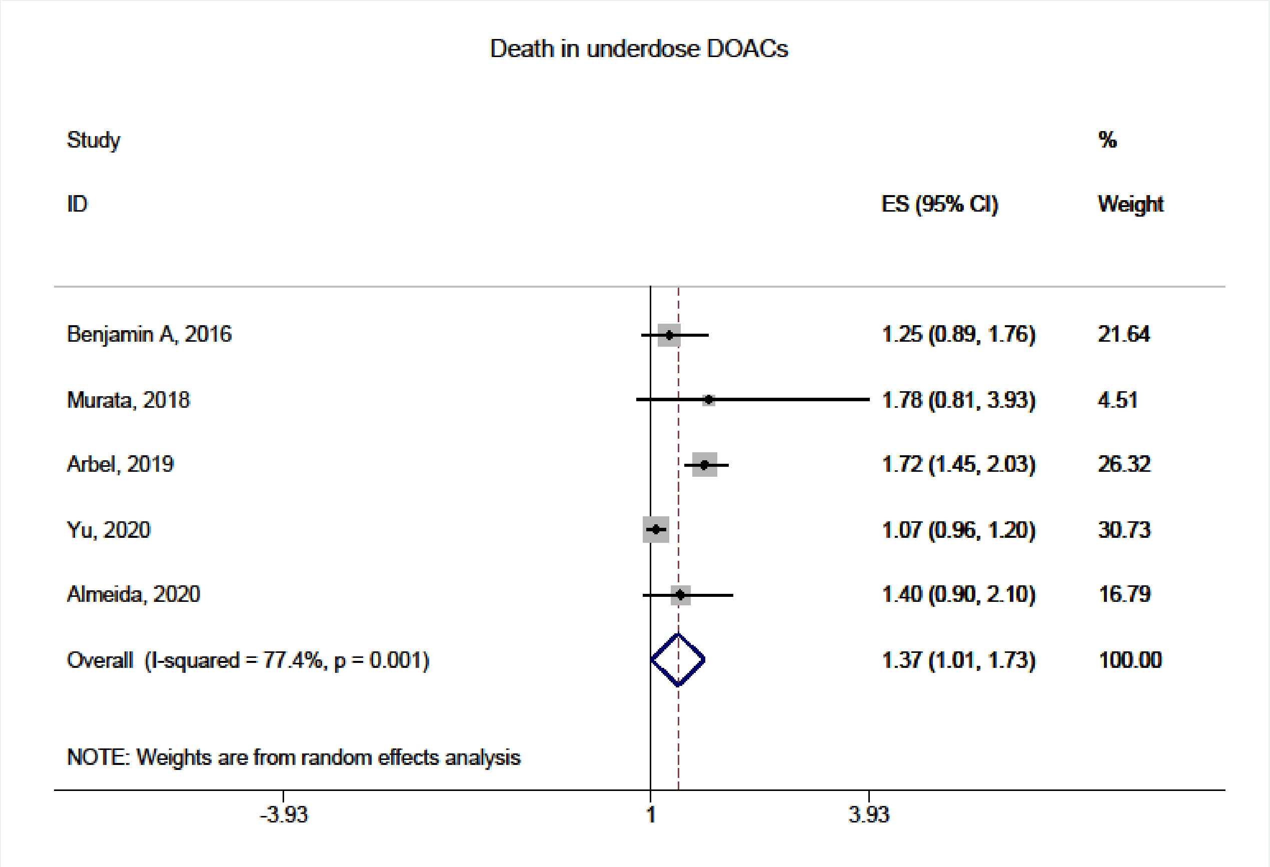

eFigure 5. Pooled Death in under-dosing of DOACs

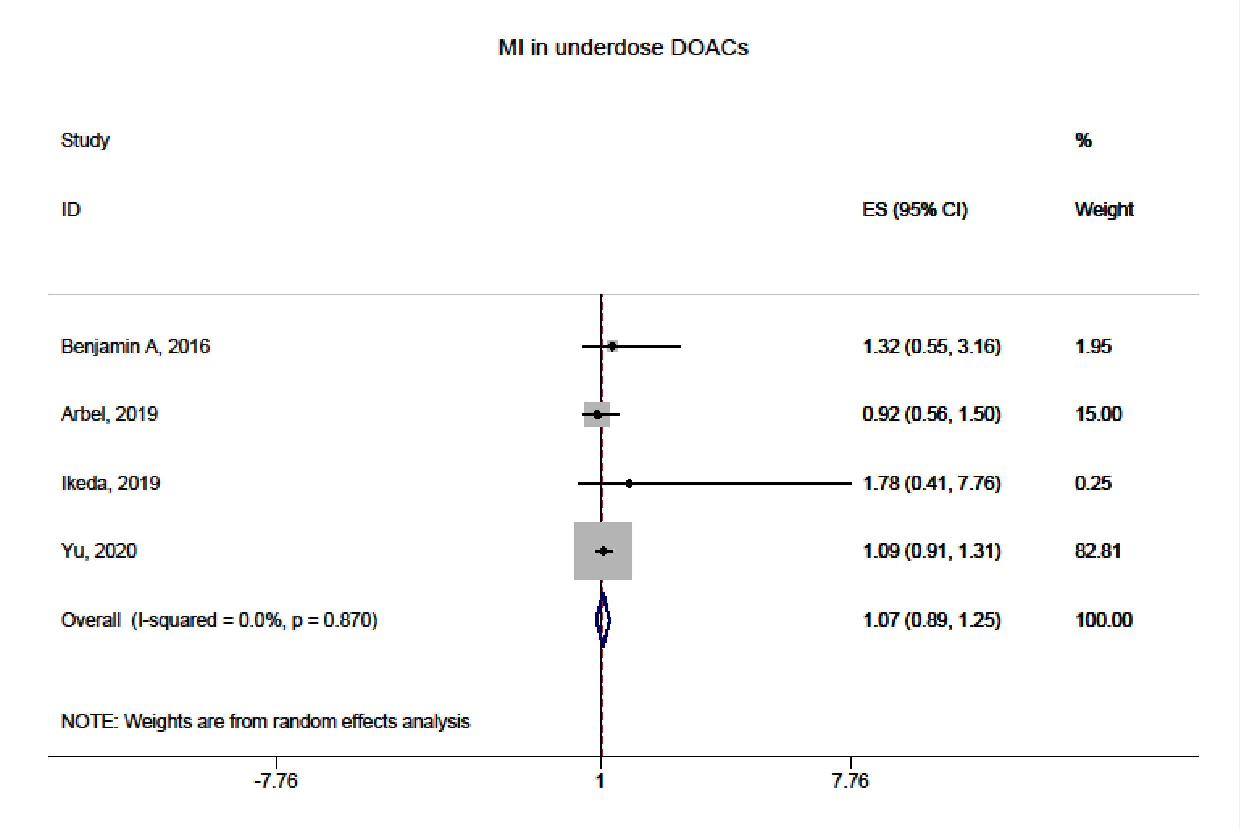

eFigure 6. Pooled MI in under-dosing of DOACs

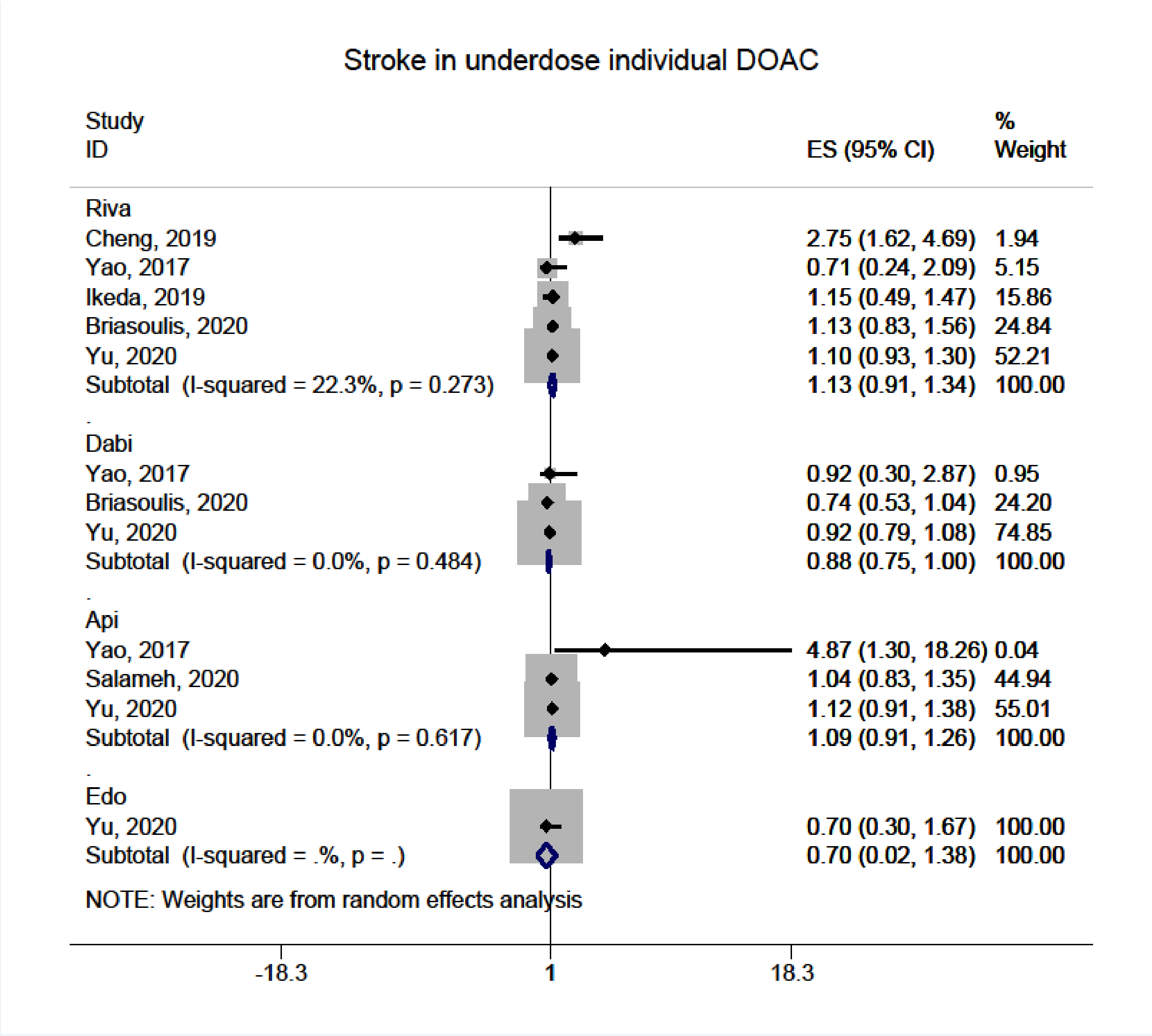

eFigure 7. Pooled stroke in under-dosing of individual DOAC

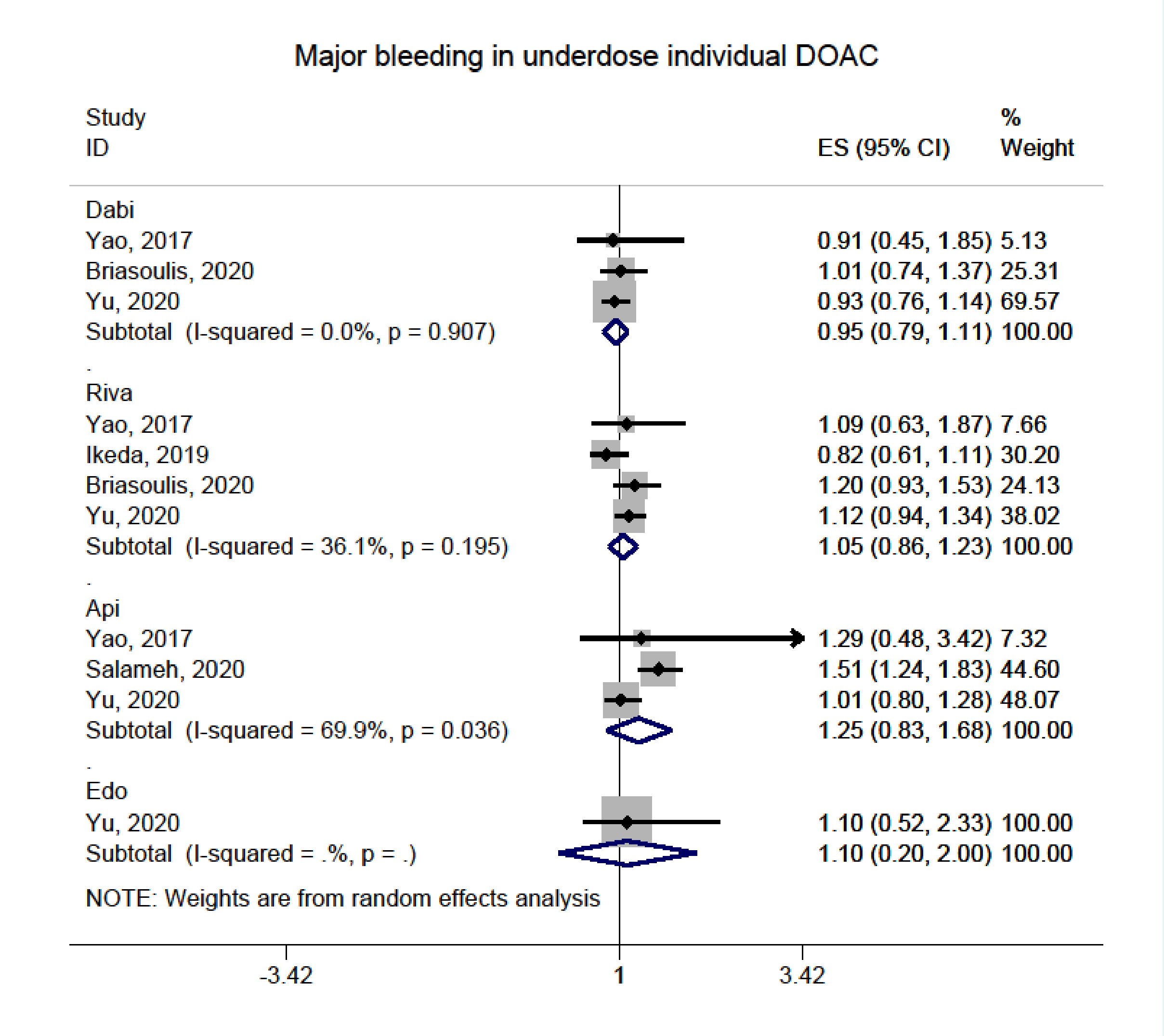

eFigure 8. Pooled major bleeding in under-dosing of individual DOAC

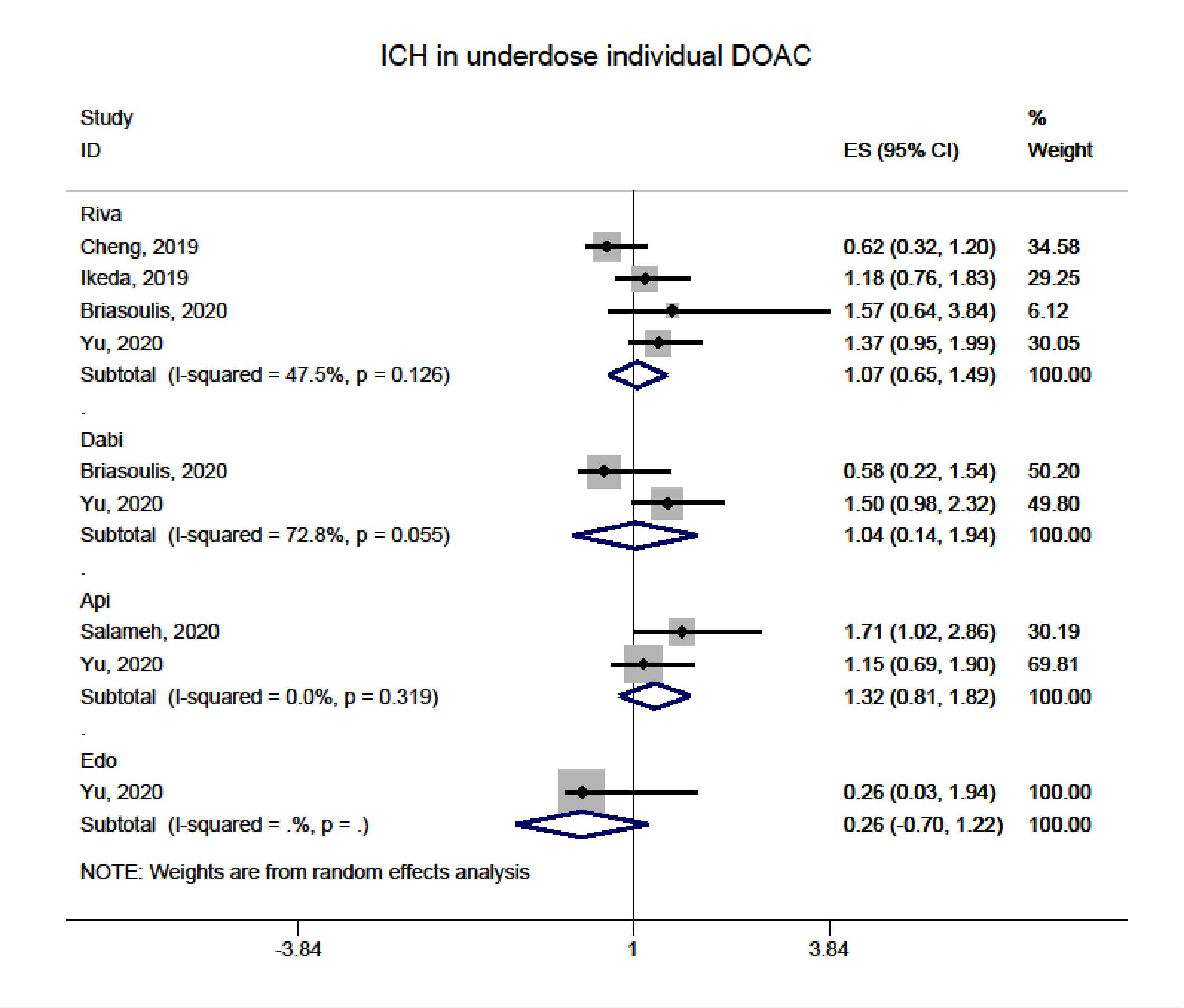

eFigure 9. Pooled ICH in under-dosing of individual DOAC

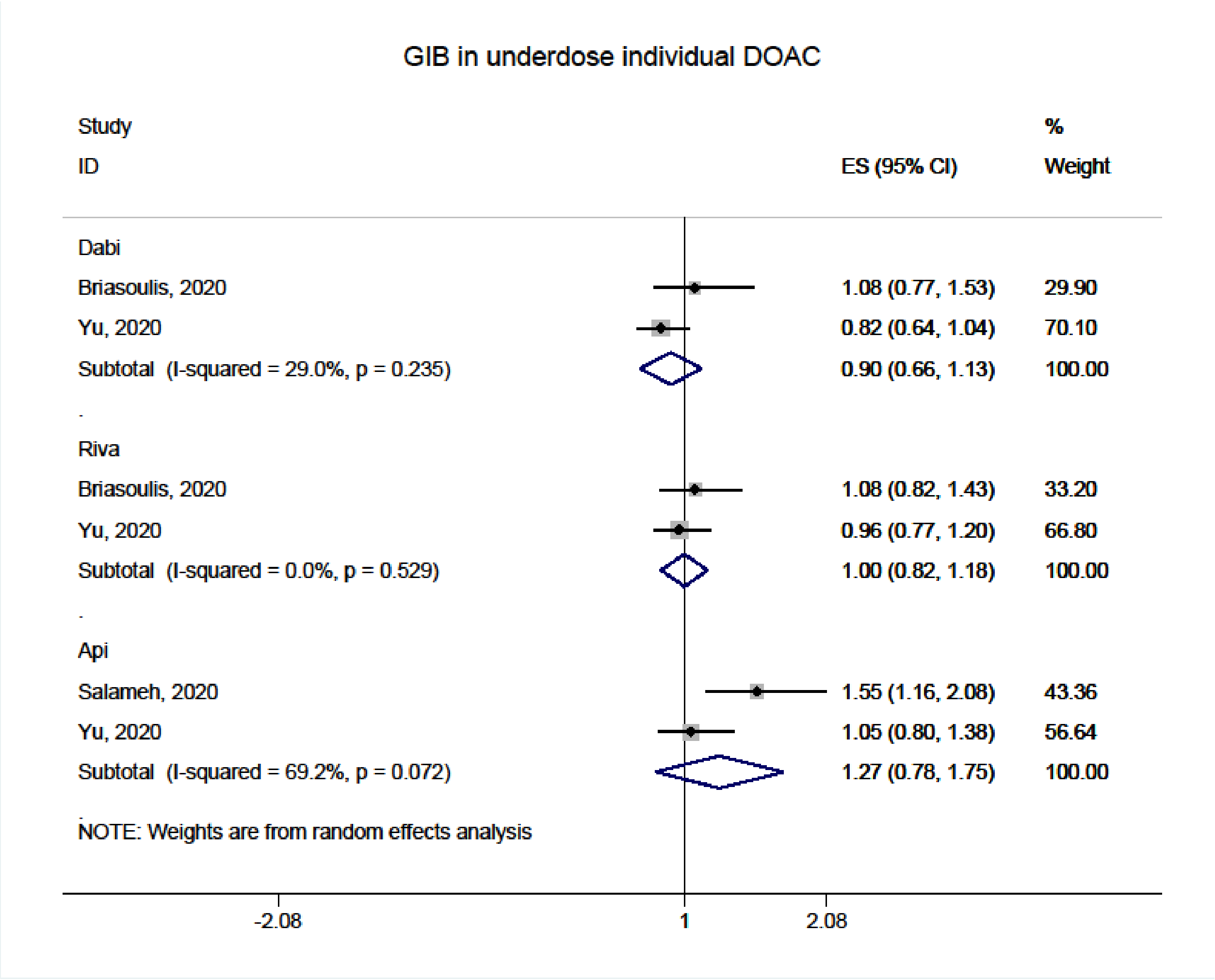

eFigure 10. Pooled GIB in under-dosing of individual DOAC

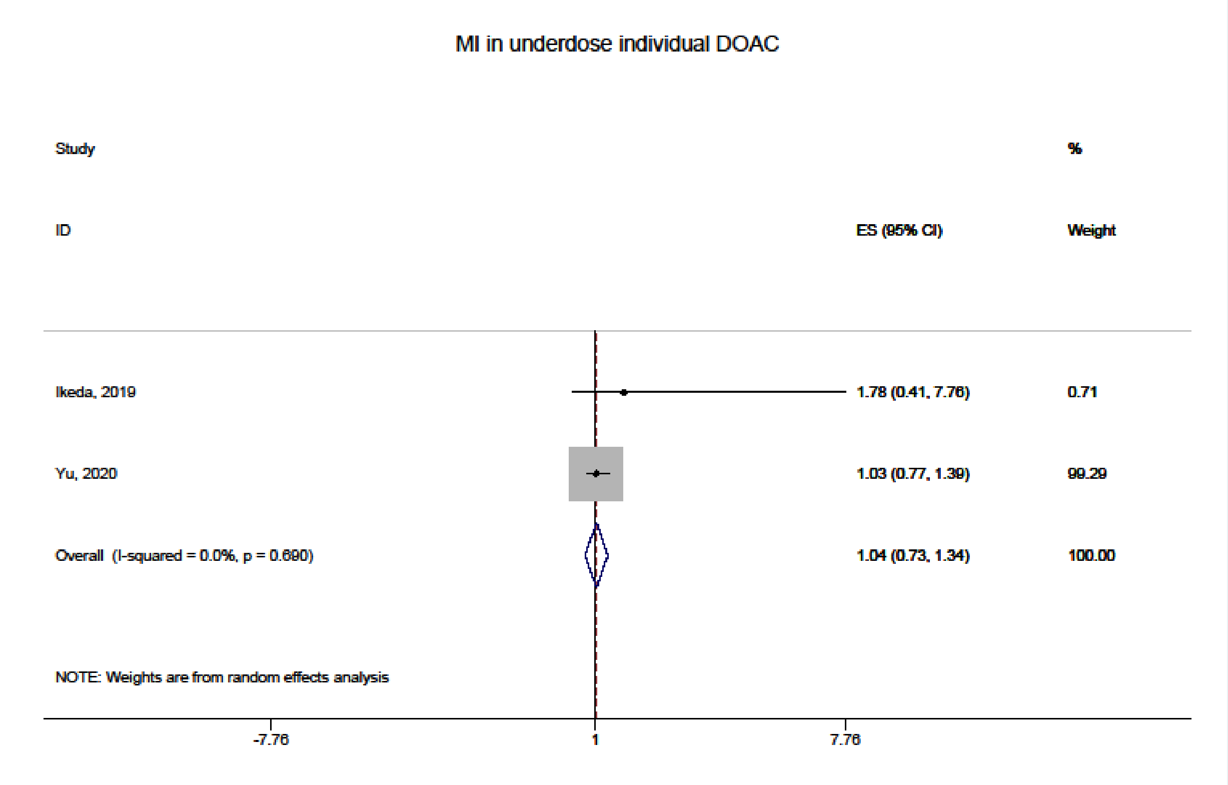

eFigure 11. Pooled MI in under-dosing of individual DOAC

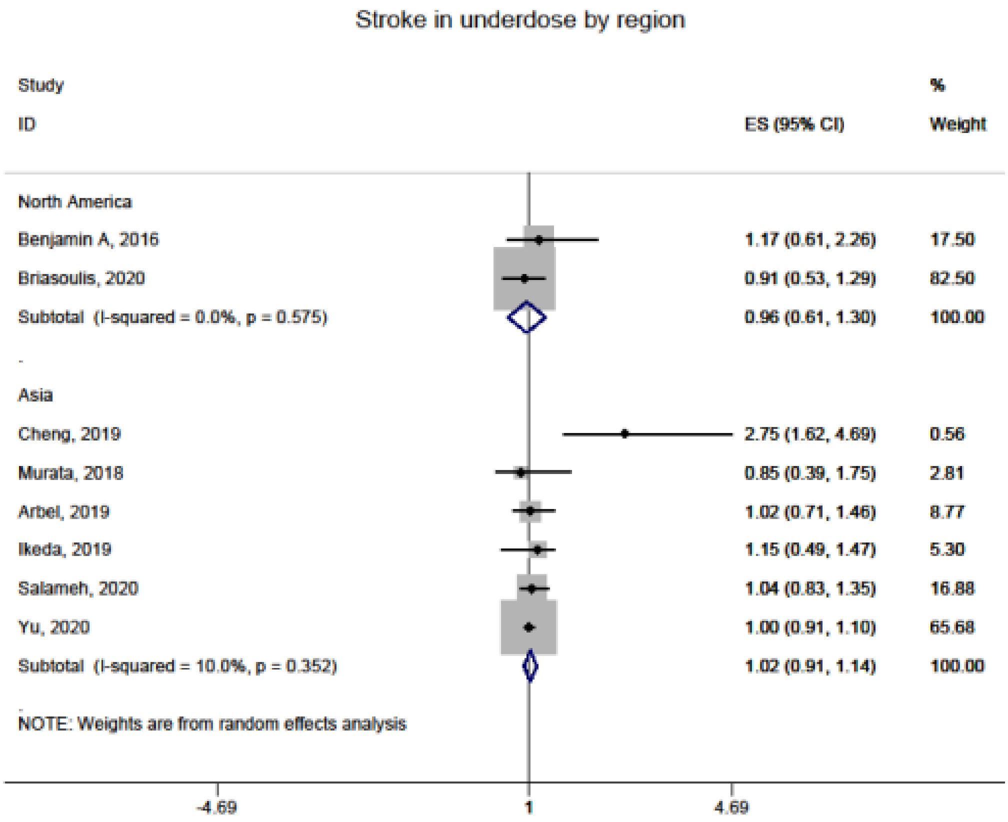

eFigure 12-1. Pooled stroke in under-dose by region

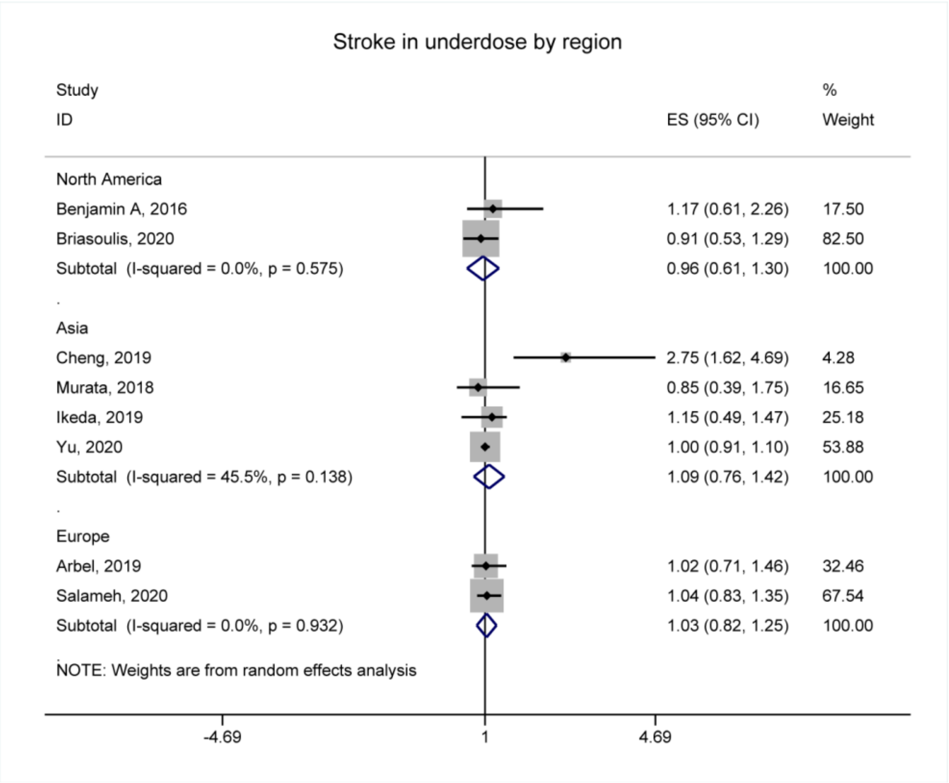

eFigure 12-2. Pooled stroke in under-dose by region (categorizing Israel as Europe)

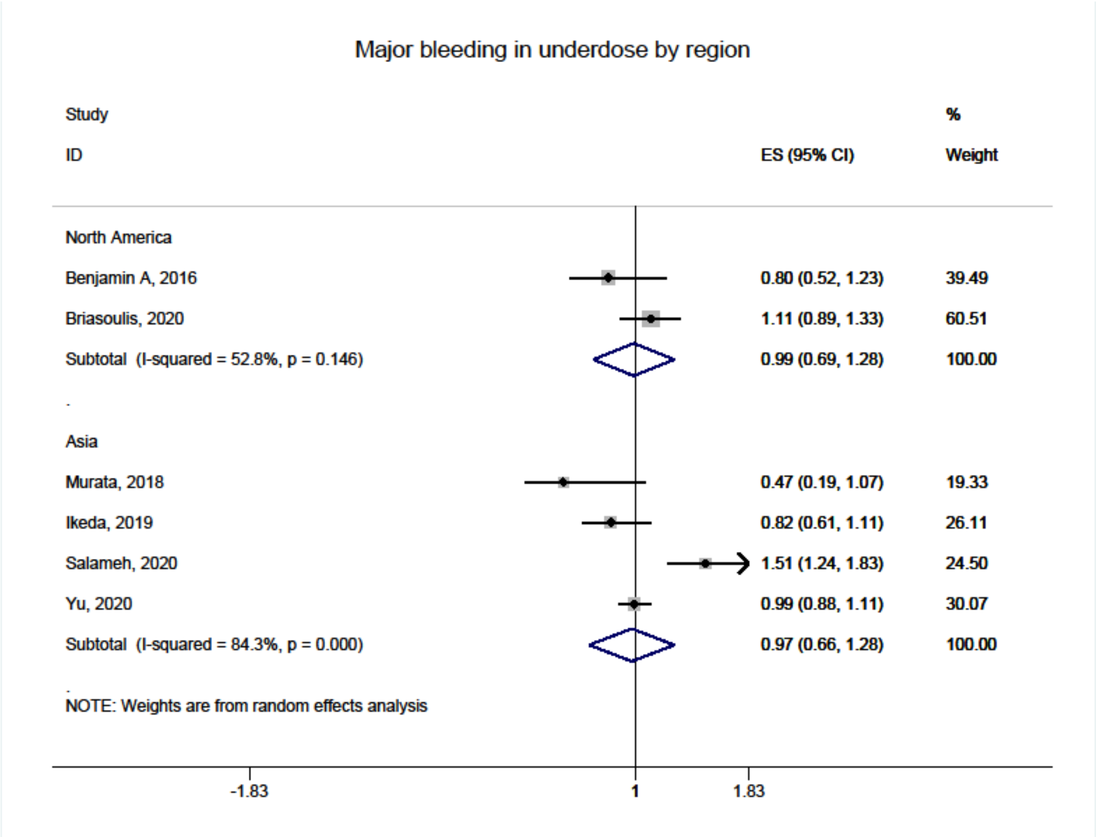

eFigure 13-1. Pooled major bleeding in under-dose by region

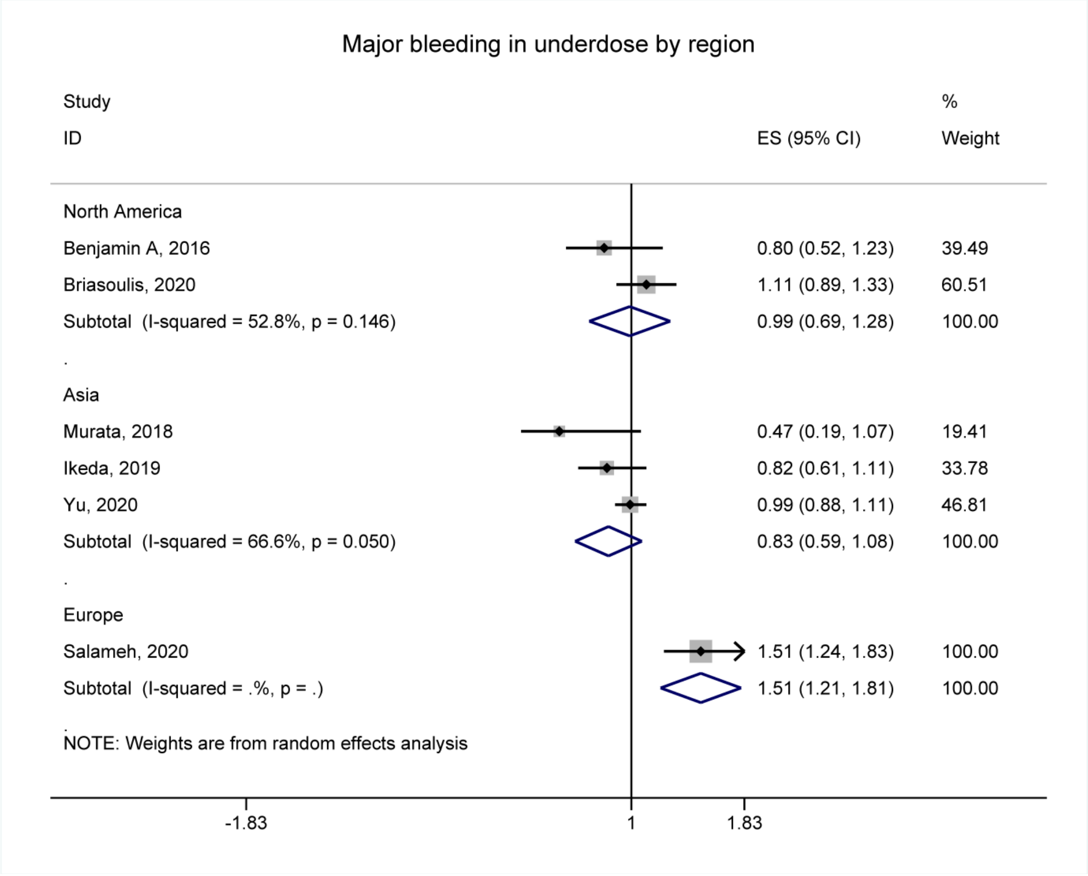

eFigure 13-2. Pooled major bleeding in under-dose by region (categorizing Israel as Europe)

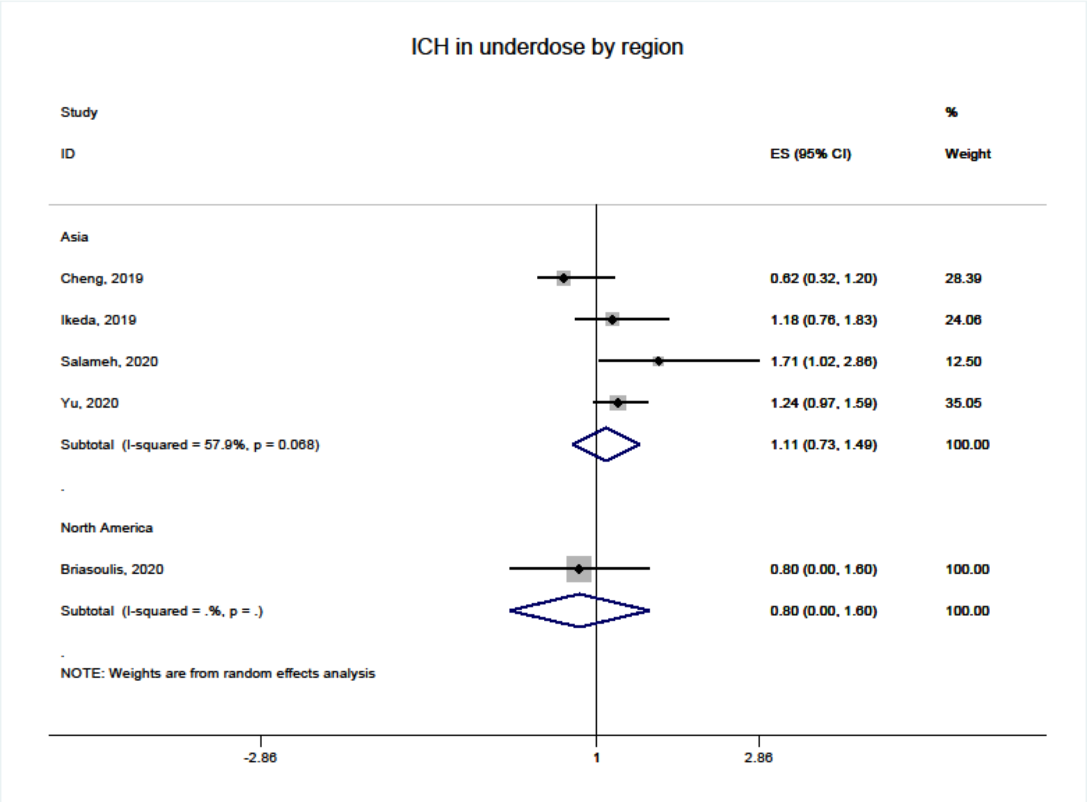

eFigure 14-1. Pooled ICH in under-dose by region

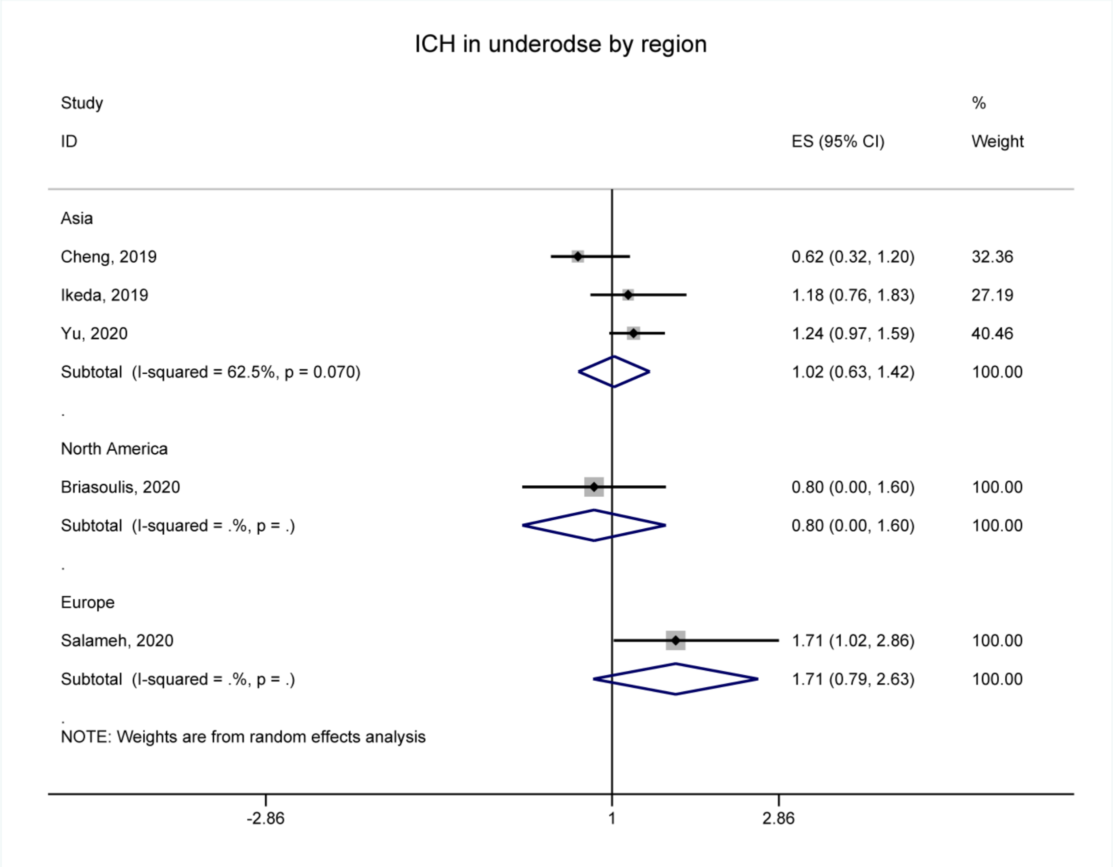

eFigure 14-2. Pooled ICH in under-dose by region (categorizing Israel as Europe)

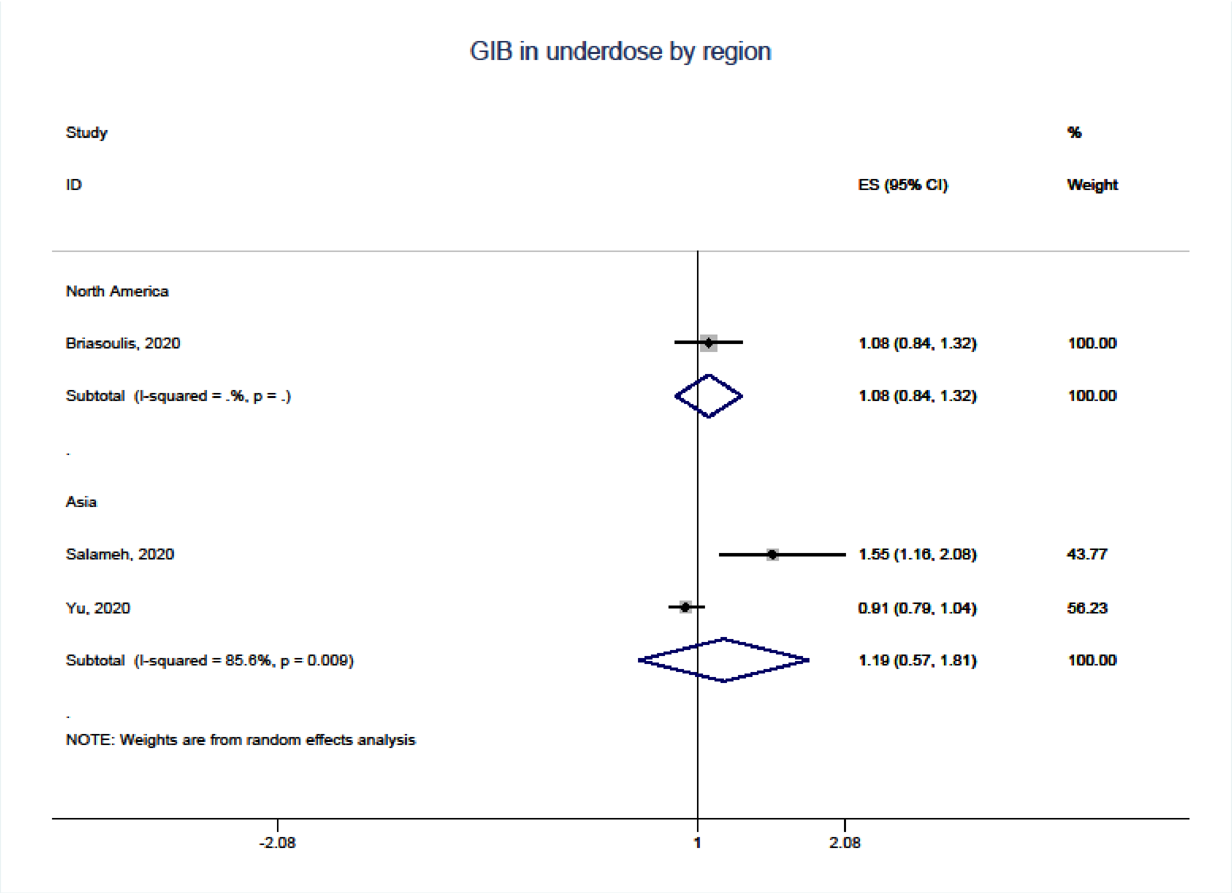

eFigure 15-1. Pooled GIB in under-dose by region

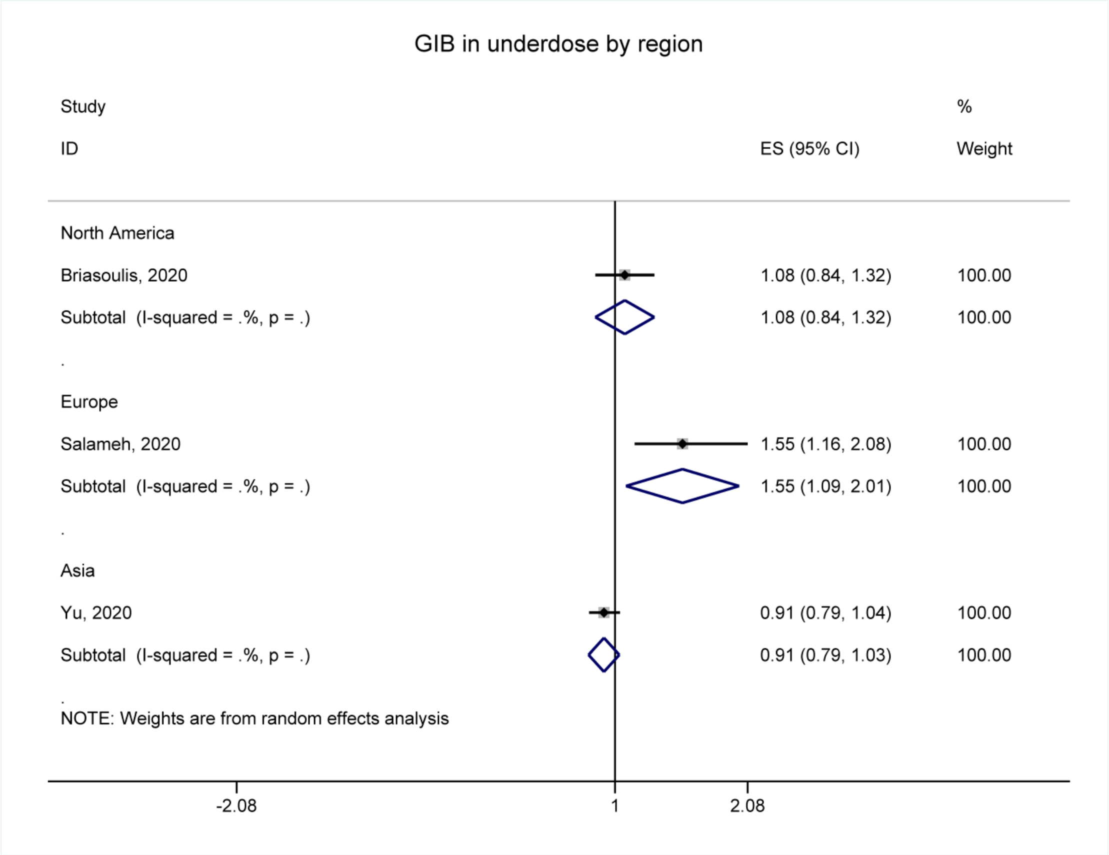

eFigure 15-2. Pooled GIB in under-dose by region (categorizing Israel as Europe)

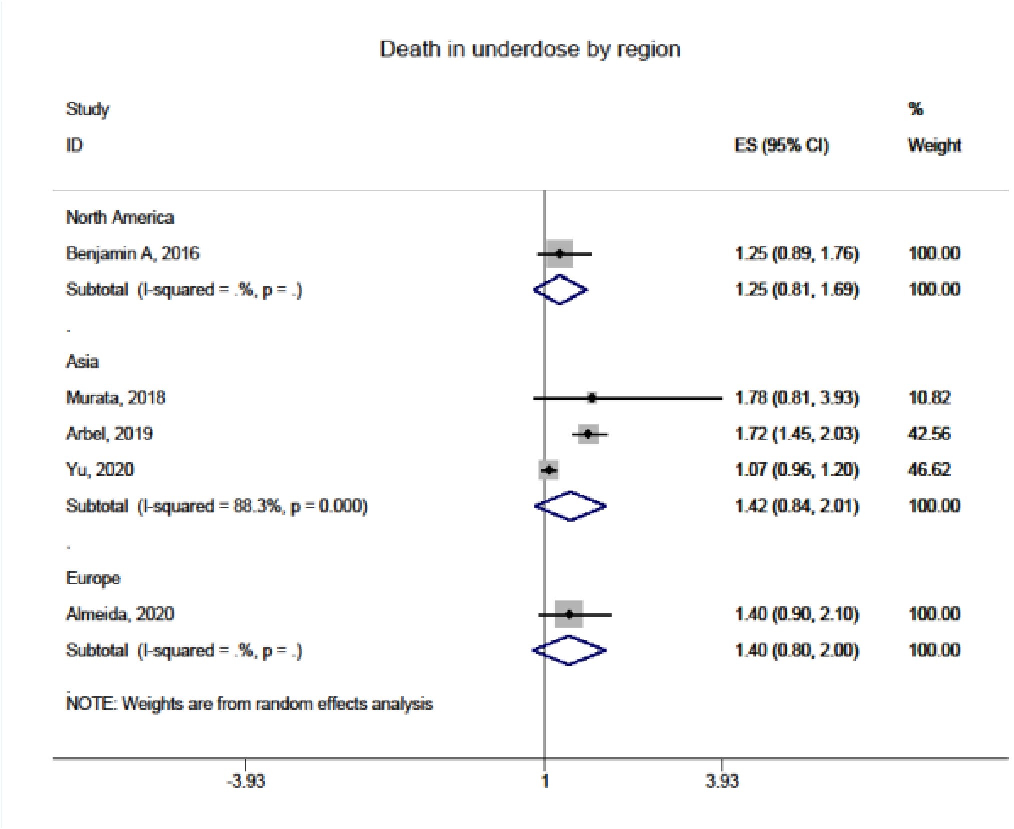

eFigure 16-1. Pooled Death in under-dose by region

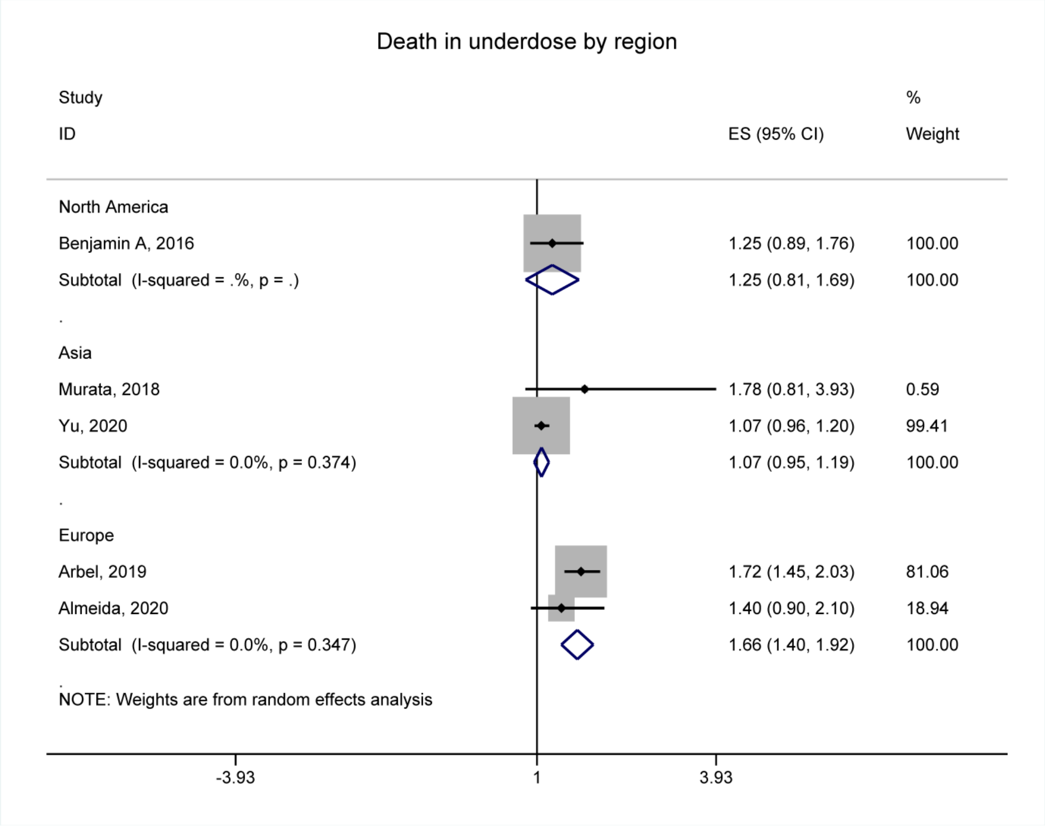

eFigure 16-2. Pooled Death in under-dose by region (categorizing Israel as Europe)

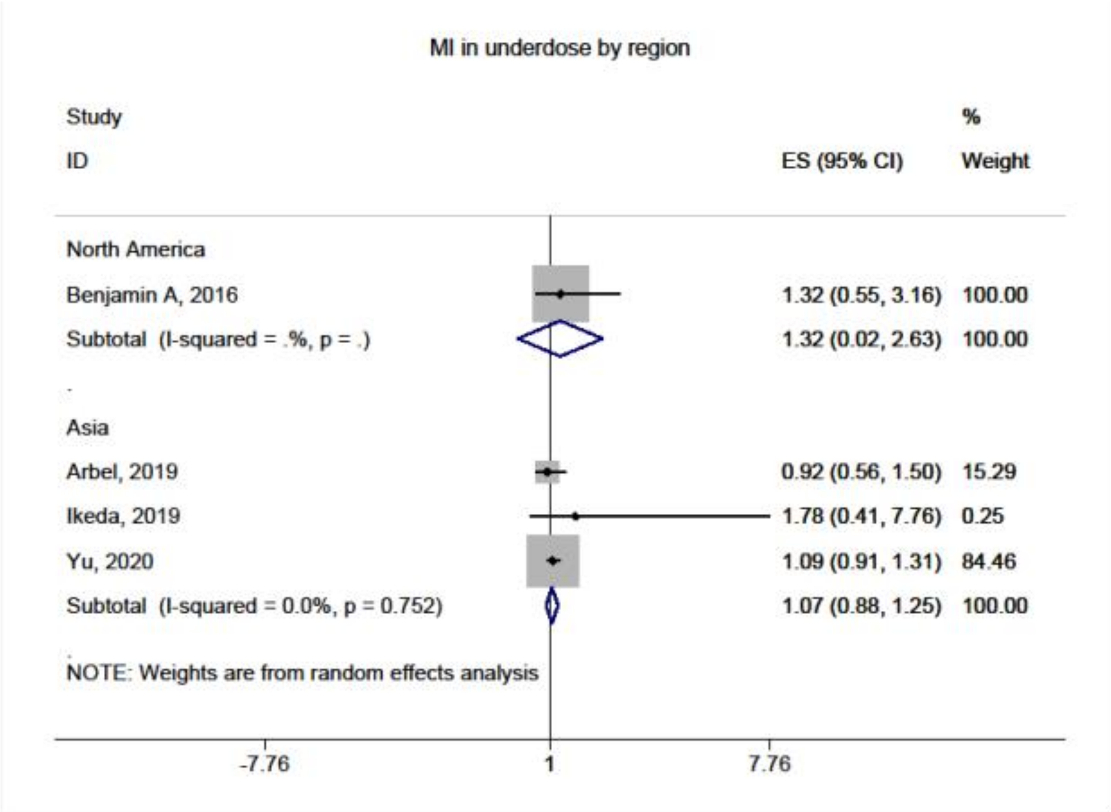

eFigure 17-1. Pooled MI in under-dose by region

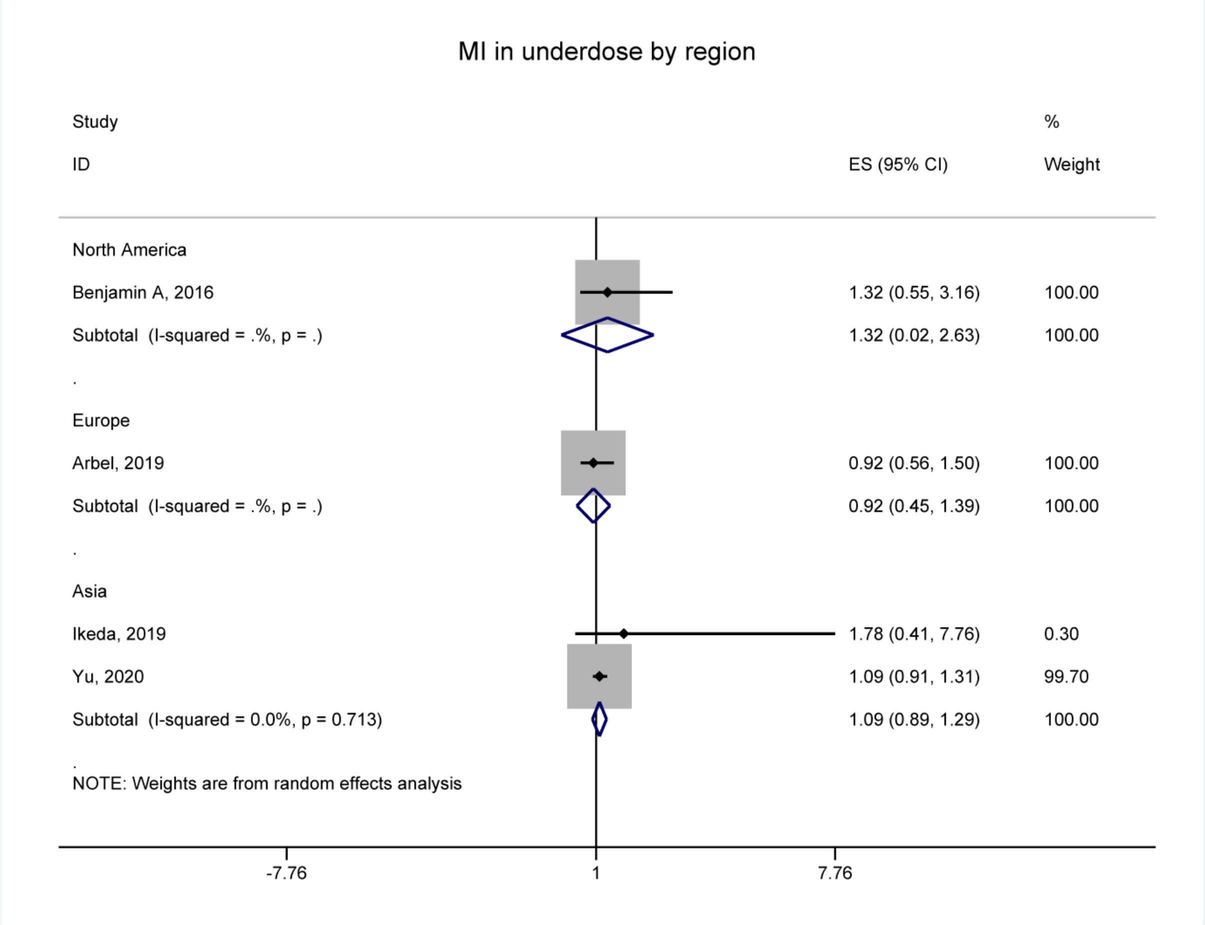

eFigure 17-2. Pooled MI in under-dose by region (categorizing Israel as Europe)

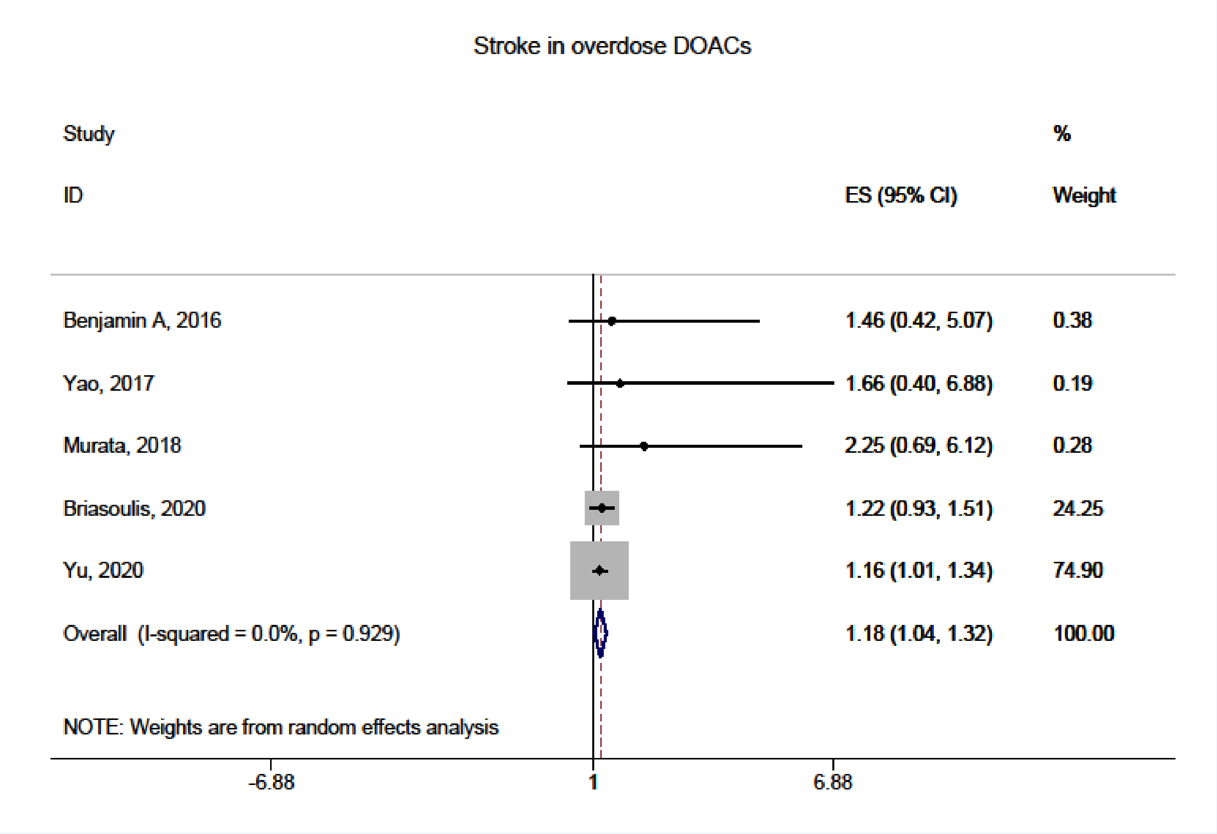

eFigure 18. Pooled stroke in over-dosing of DOACs

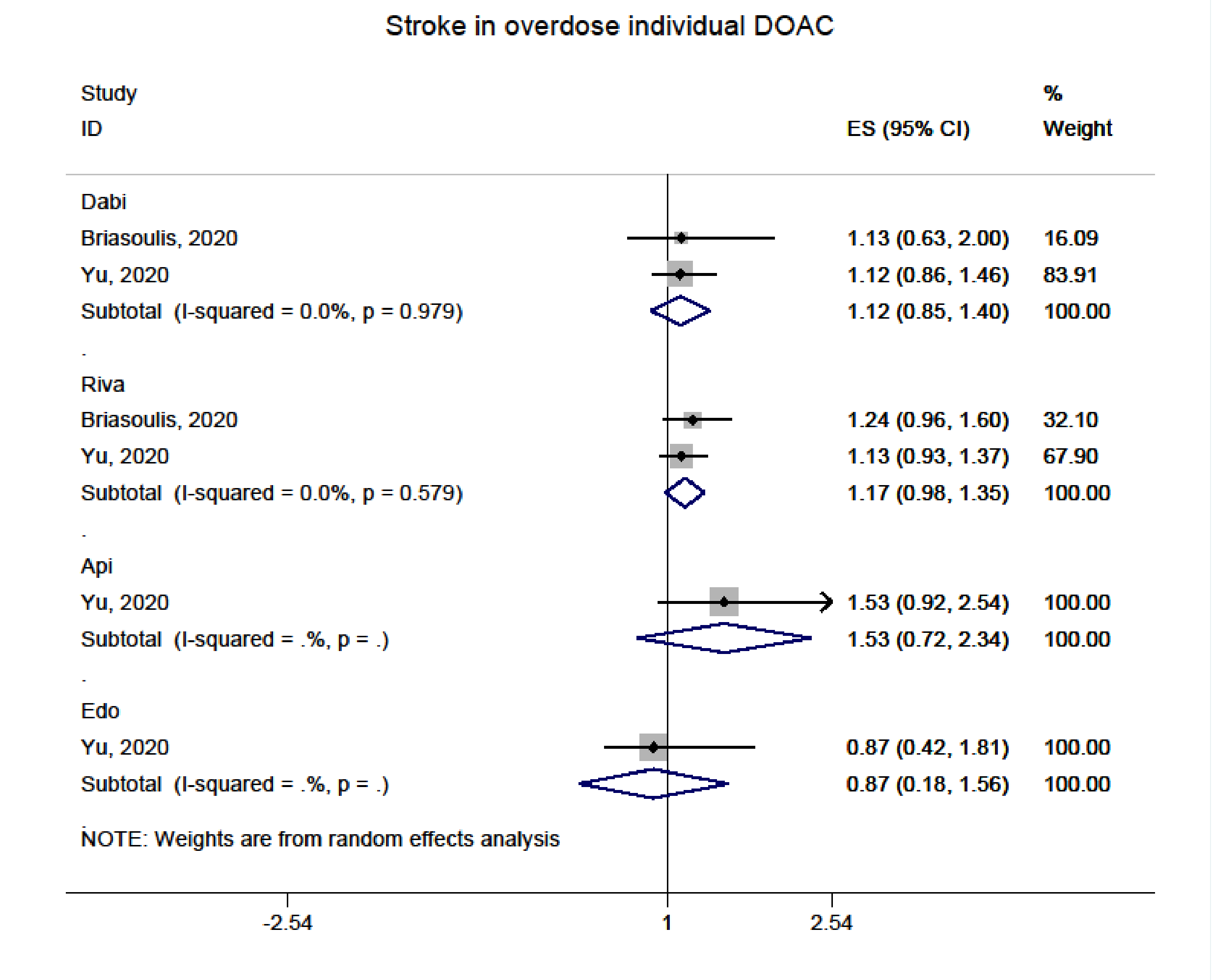

eFigure 19. Pooled stroke in over-dosing of individual DOAC

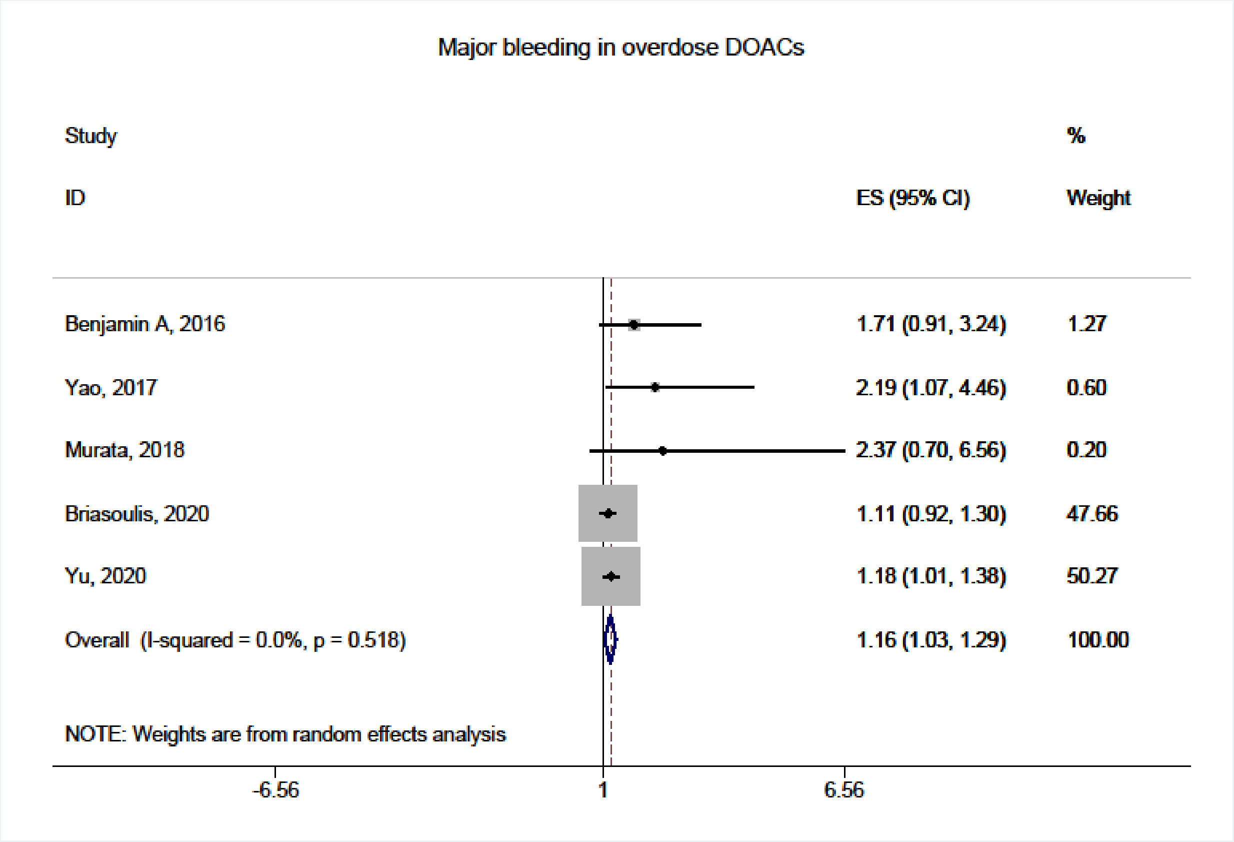

eFigure 20. Pooled major bleeding in over-dosing of DOACs

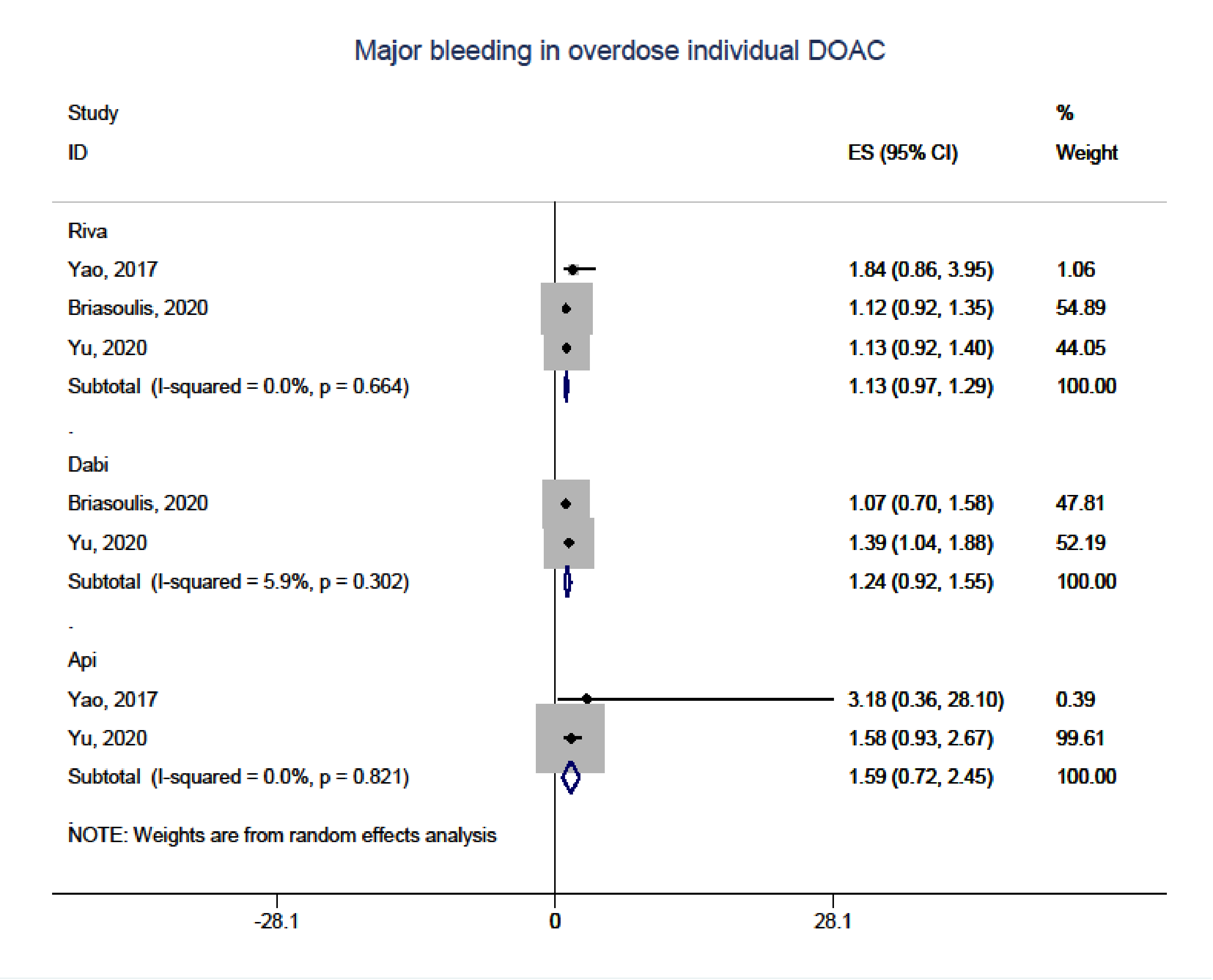

eFigure 21. Pooled major bleeding in over-dosing of individual DOAC

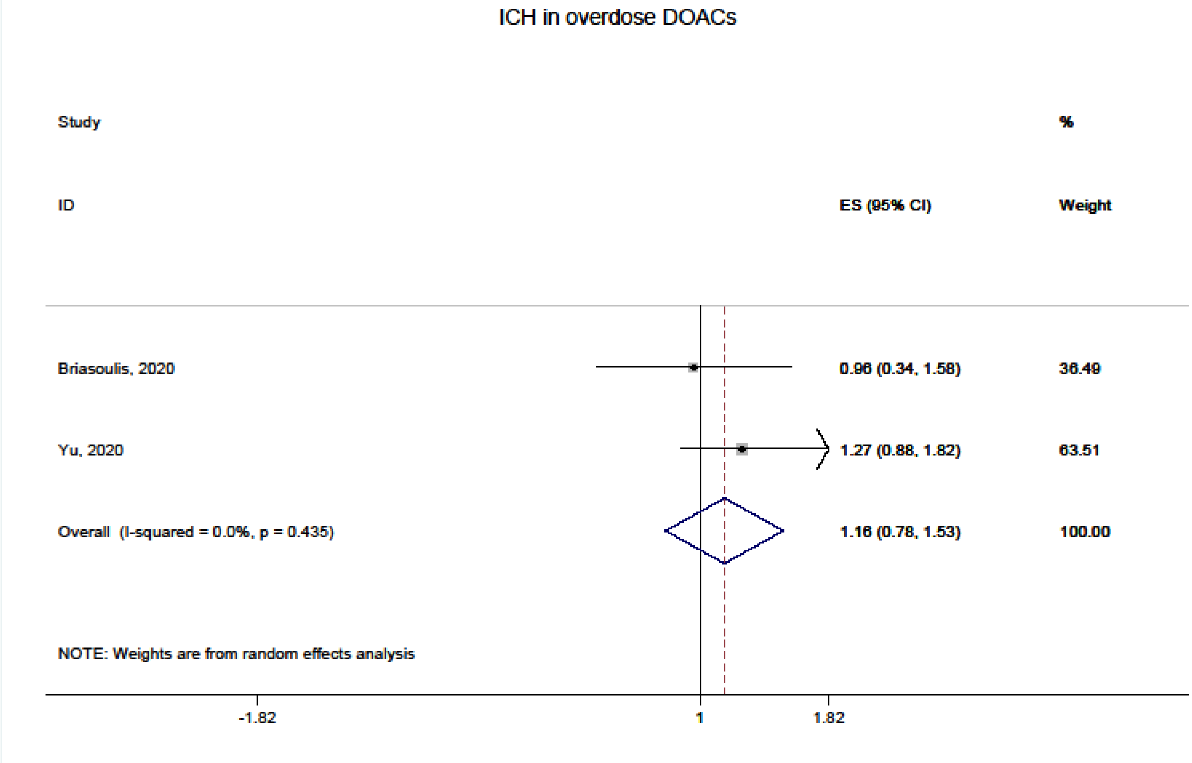

eFigure 22. Pooled ICH in over-dosing of DOACs

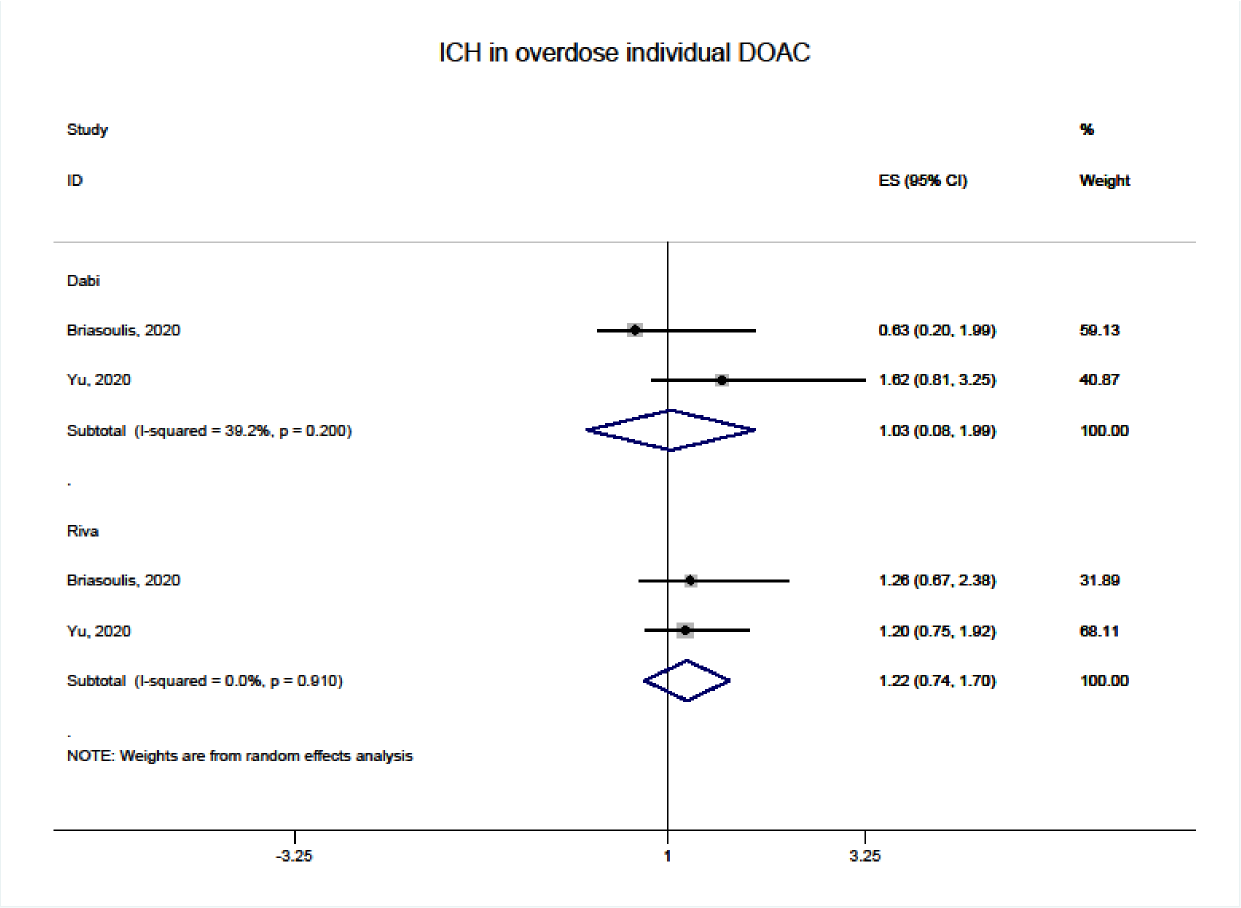

eFigure 23. Pooled ICH in over-dosing of individual DOAC

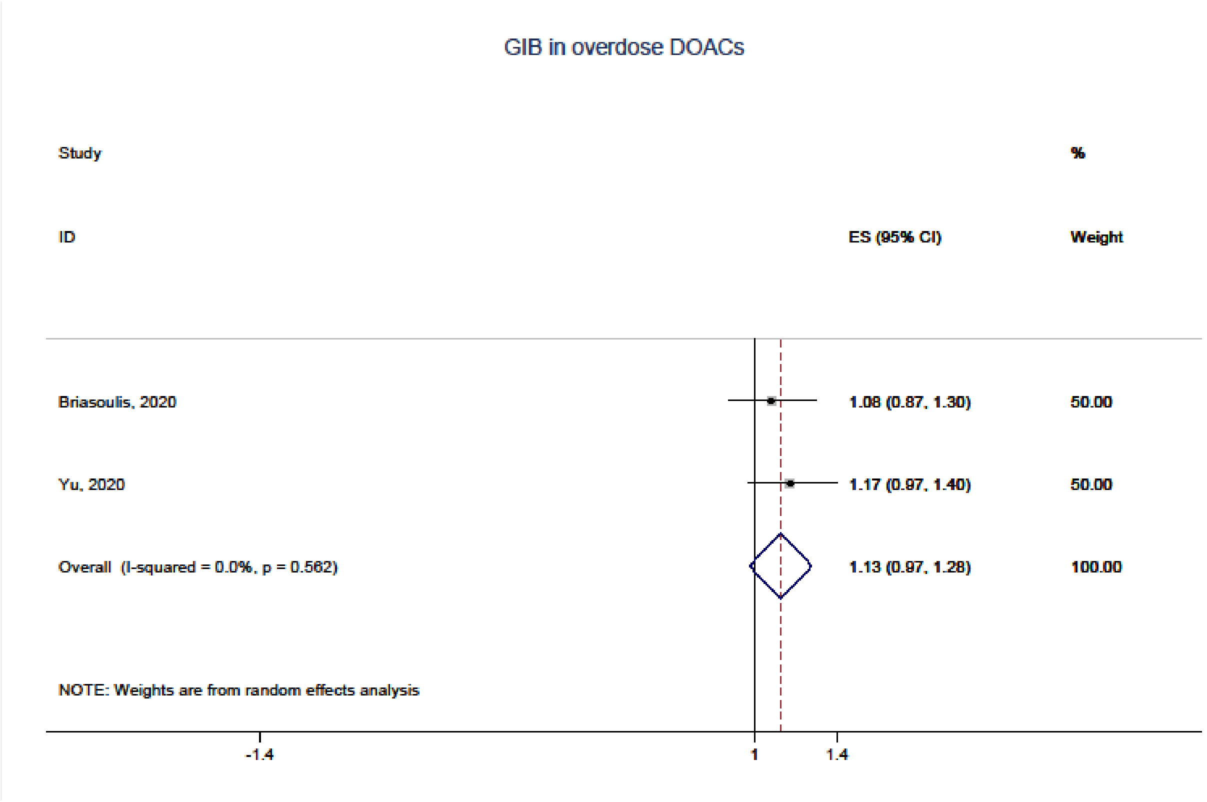

eFigure 24. Pooled GIB in over-dosing of DOACs

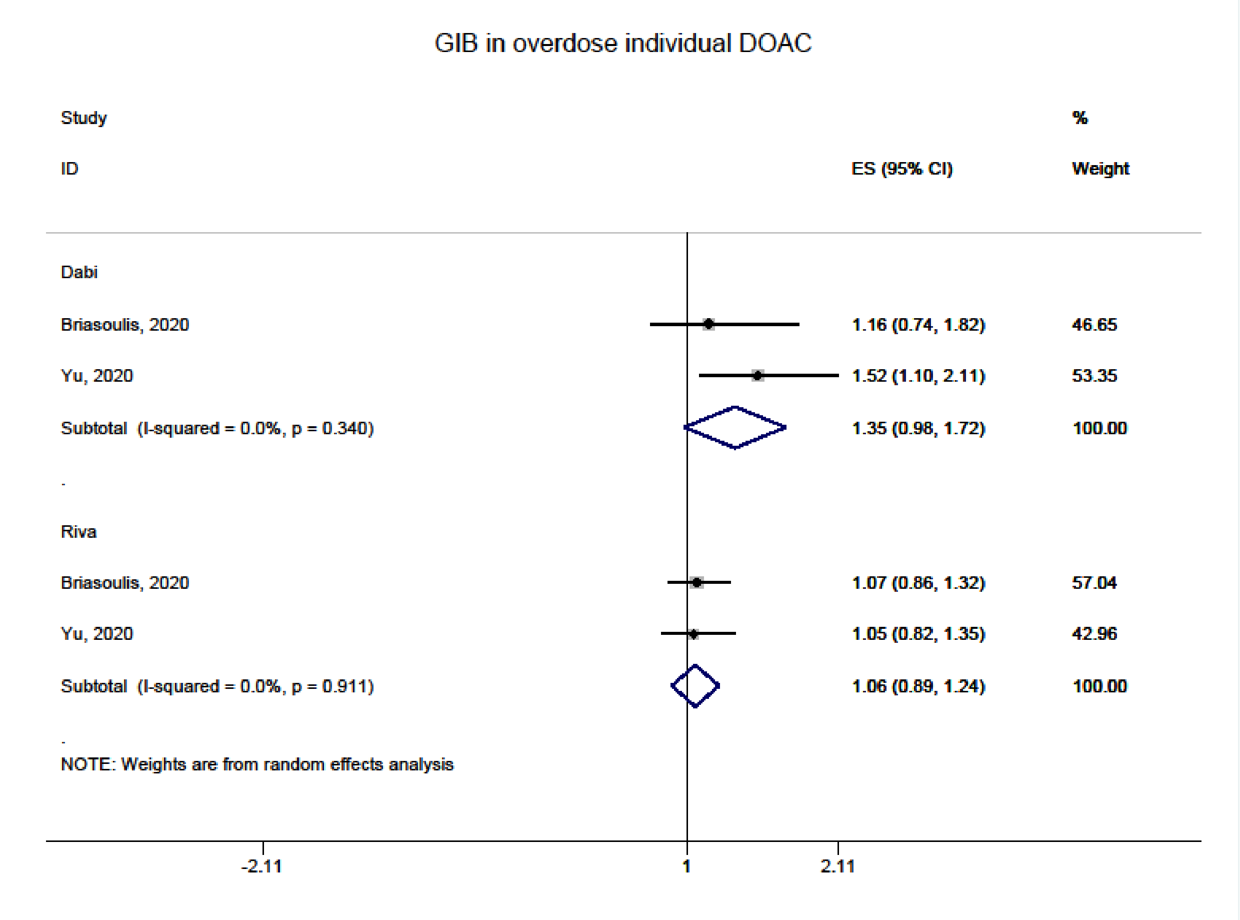

eFigure 25. Pooled GIB in over-dosing of individual DOAC

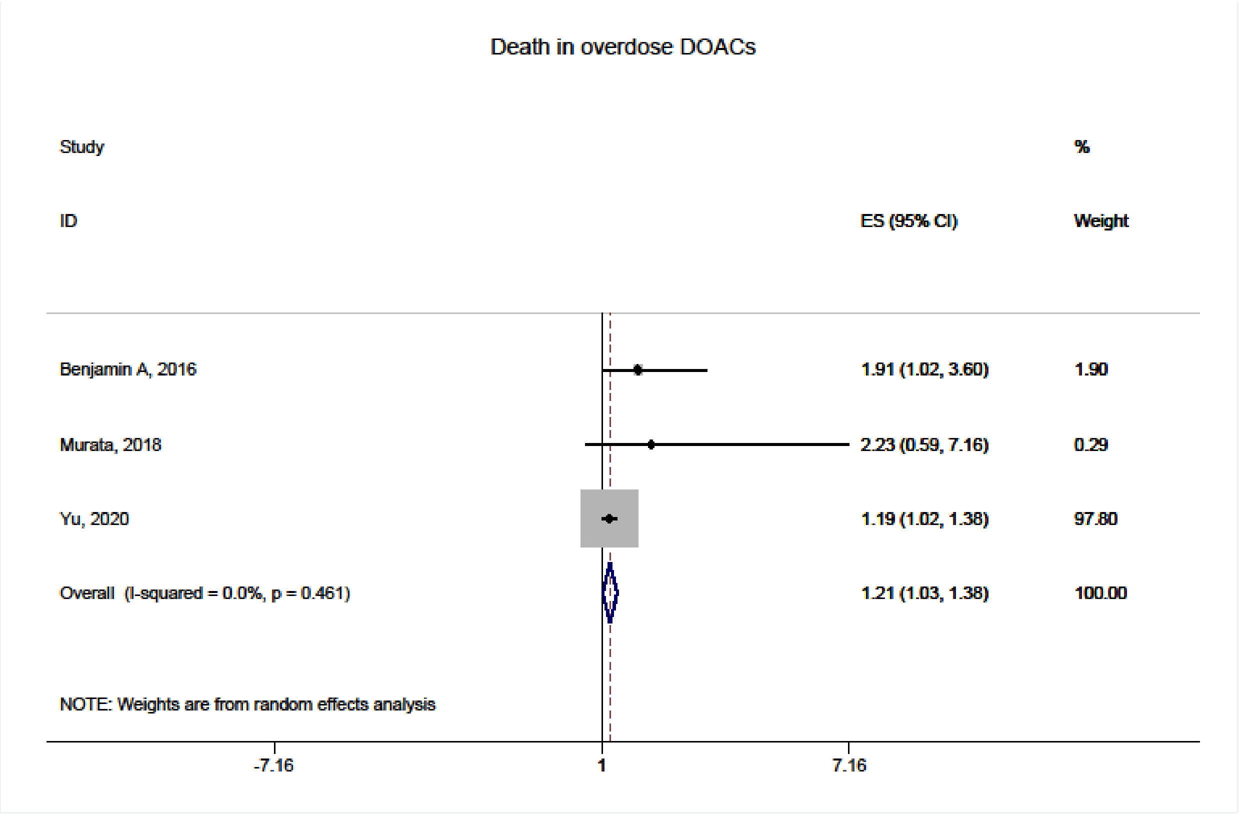

eFigure 26. Pooled Death in over-dosing of DOACs

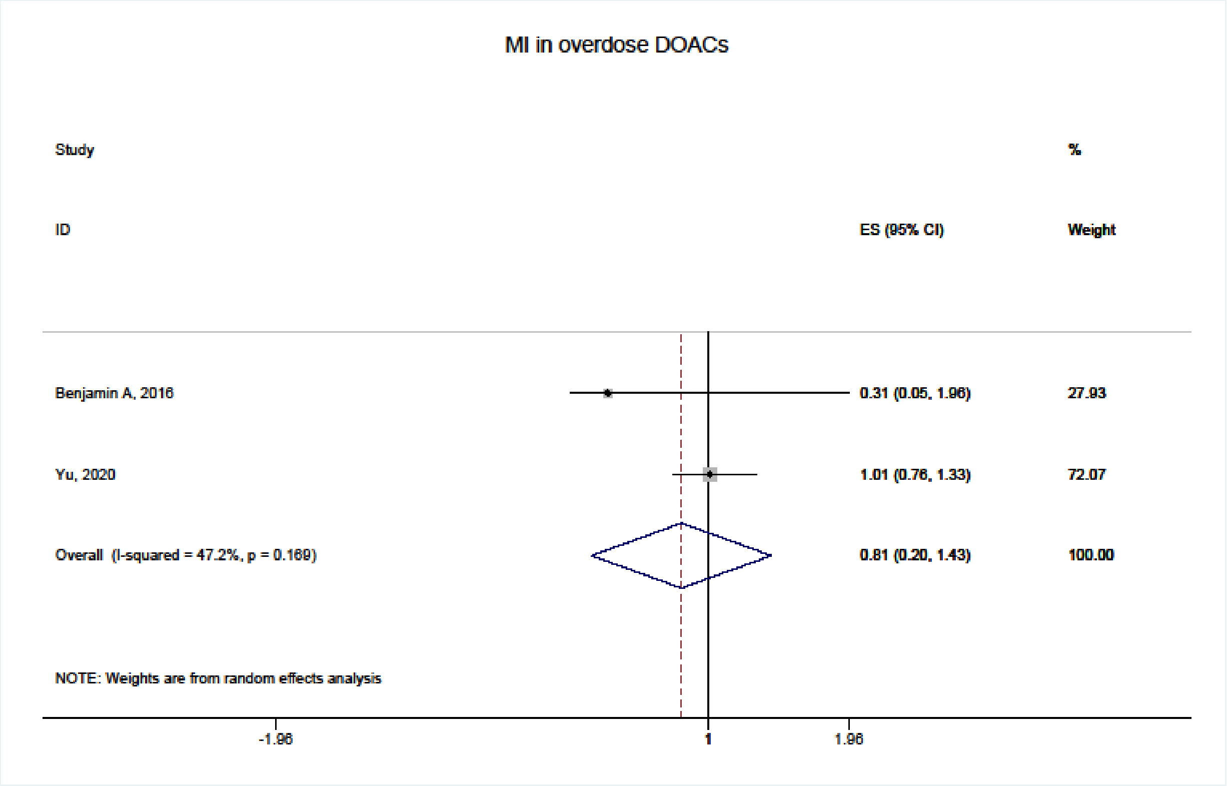

eFigure 27. Pooled MI in over-dosing of DOACs

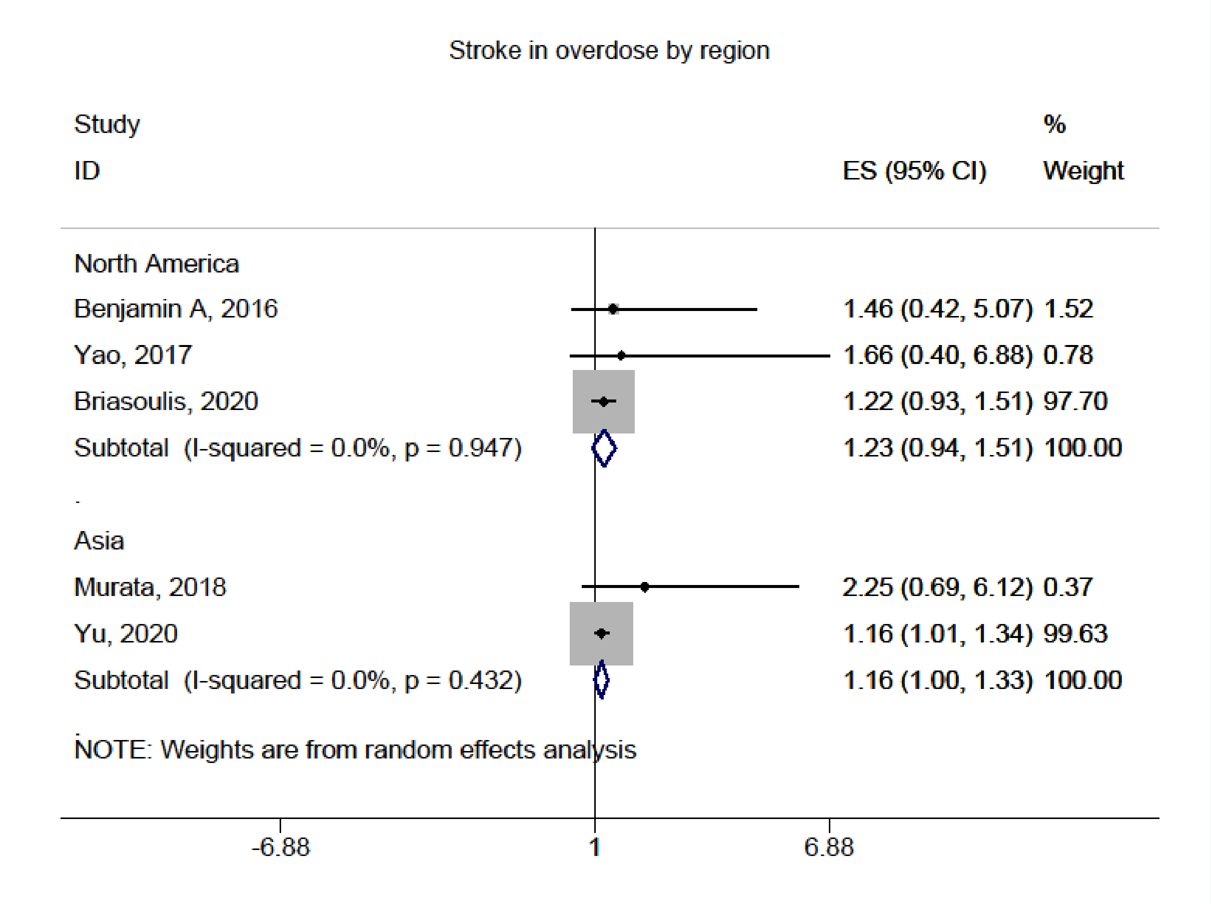

eFigure 28. Pooled stroke in over-dose by region

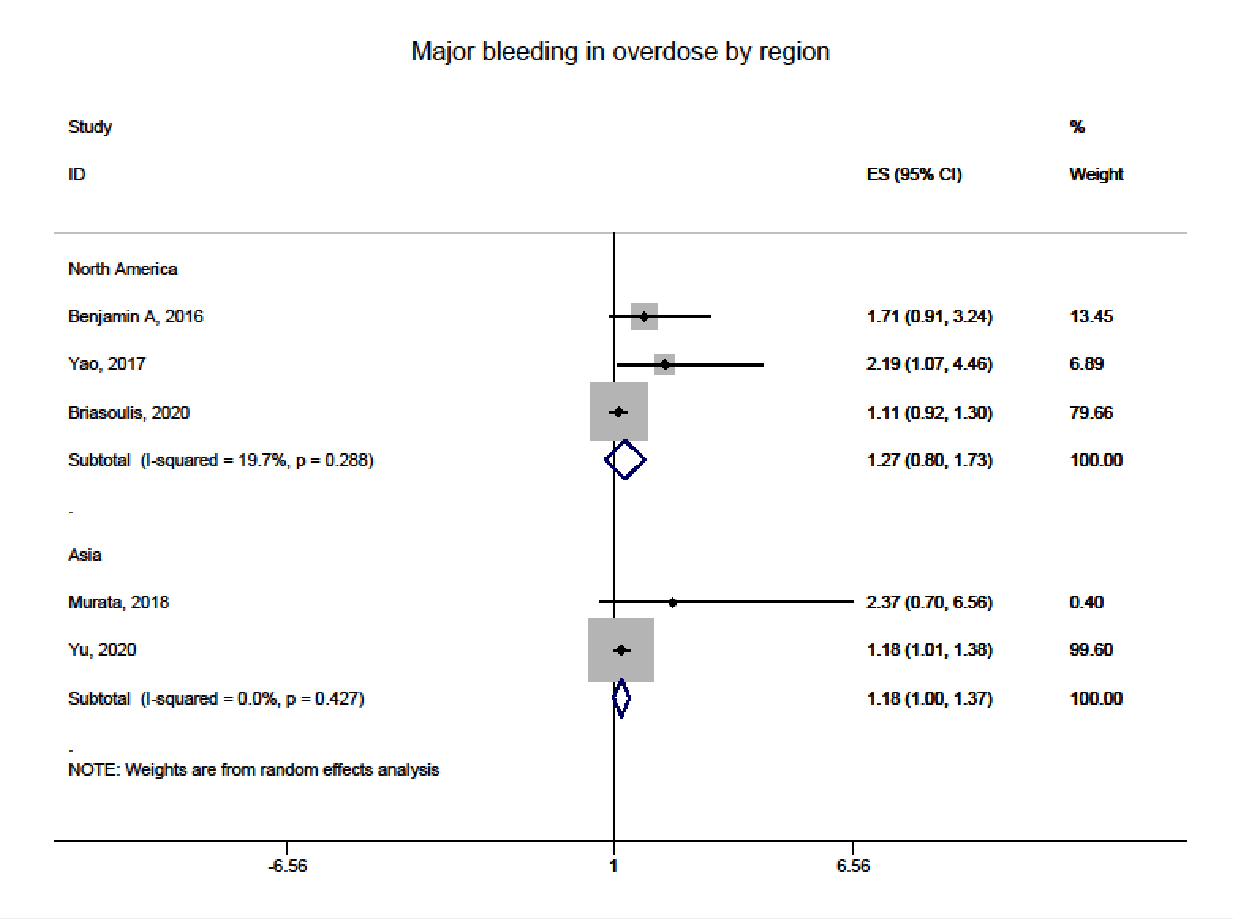

eFigure 29. Pooled major bleeding in over-dose by region

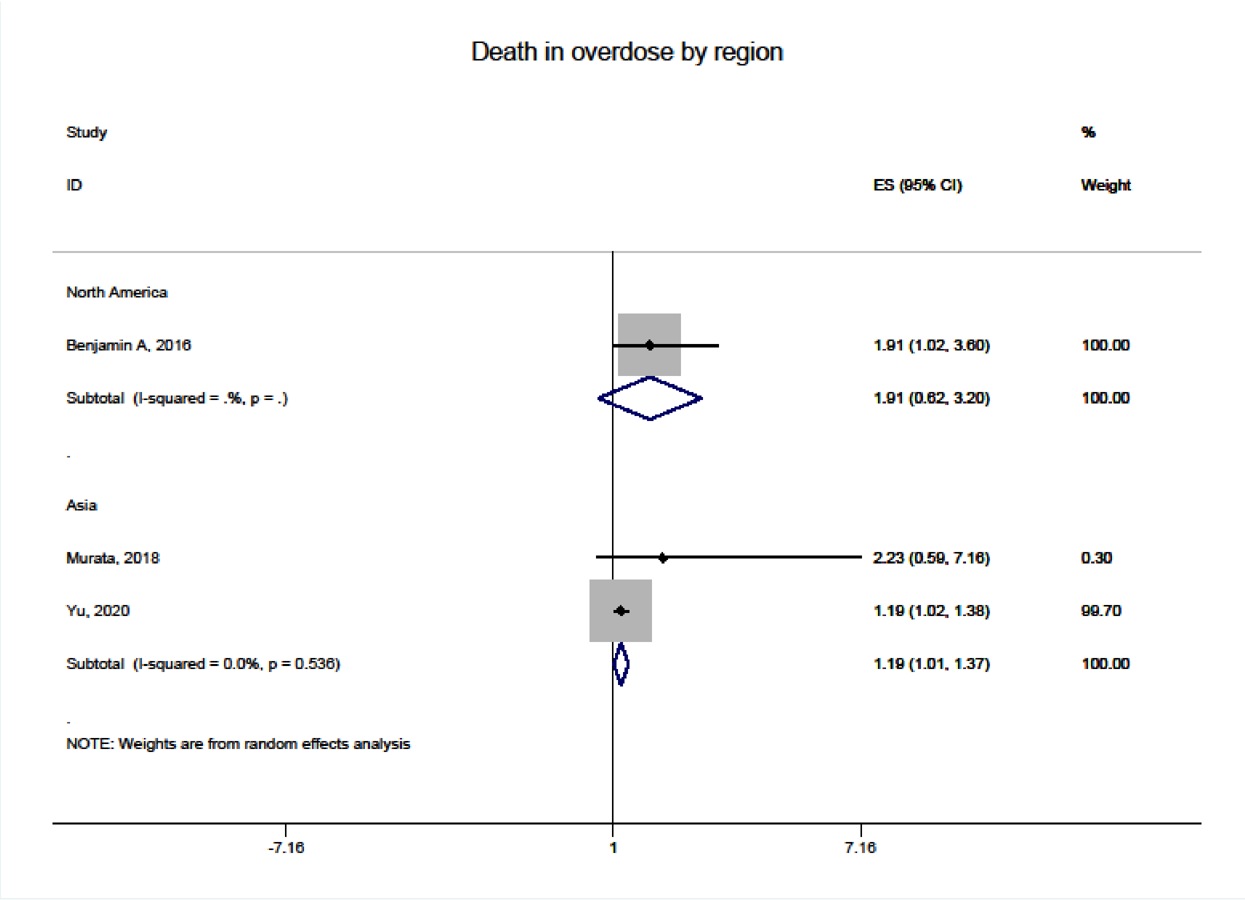

eFigure 30. Pooled Death in over-dose by region

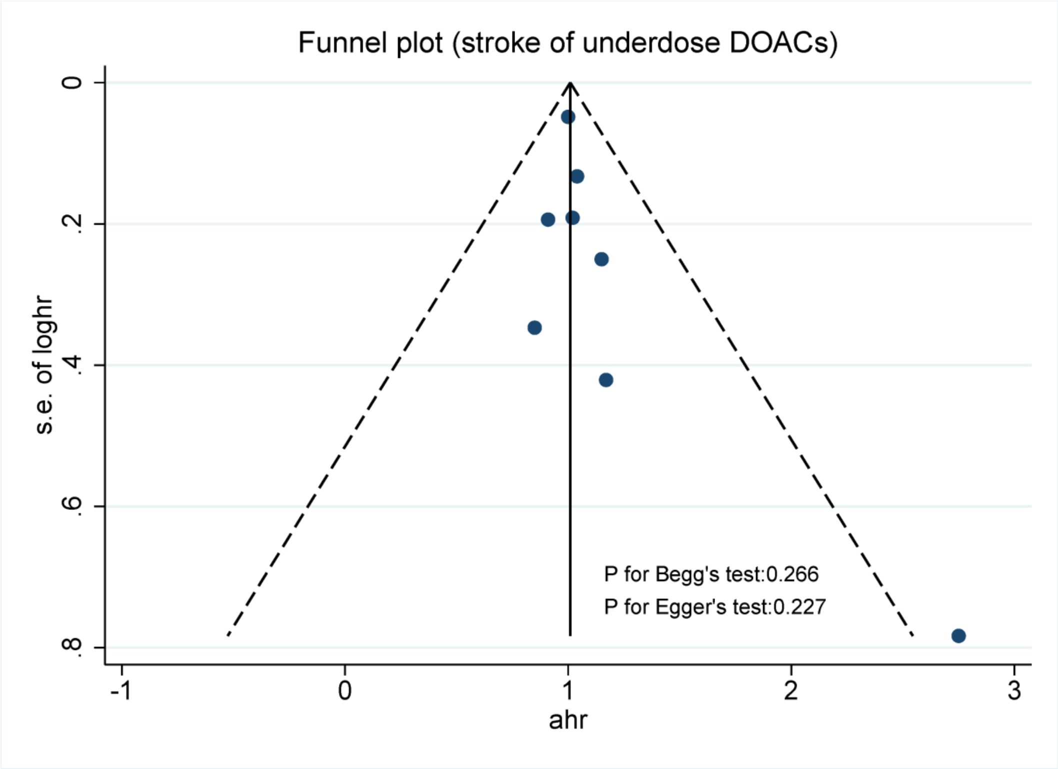

eFigure 31. Funnel plot (stroke of under-dosing of DOACs)

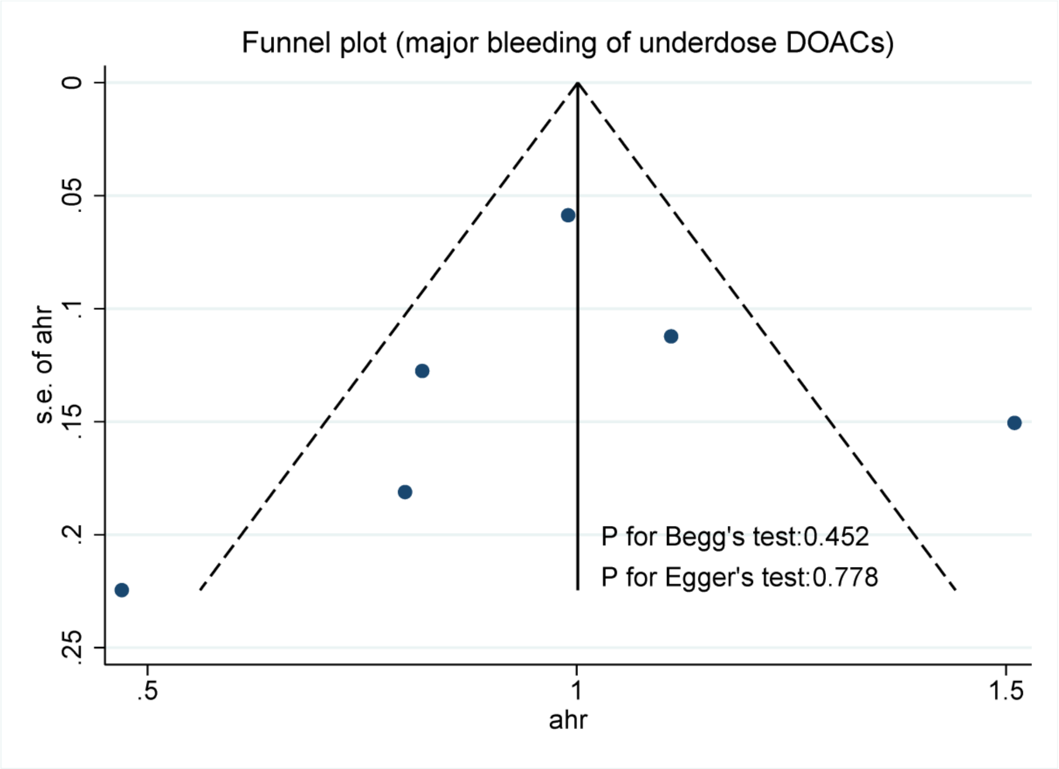

eFigure 32. Funnel plot (major bleeding of under-dosing of DOACs)

References

Ablefoni, K., and Buchholz, A. (2019). Initial rivaroxaban dosing in patients with atrial fibrillation. 42(10), 873-880. doi: 10.1080/03007995.2019.1647735

10.1002/clc.23235.

Alali, N., Mahmoud, M.H., Alharbi, M.A., and Ghazal, S.N. (2019). Appropriateness of dabigatran dosing in patients with nonvalvular atrial fibrillation (NVAF): A retrospective study conducted in a tertiary care university hospital in the eastern province of Saudi Arabia. *J Saudi Heart Assoc* 31(3), 130-134. doi: 10.1111/sdi.12829

10.1016/j.jsha.2019.05.002.

Barra, M.E., Fanikos, J., Connors, J.M., Sylvester, K.W., Piazza, G., and Goldhaber, S.Z. (2016). Evaluation of Dose-Reduced Direct Oral Anticoagulant Therapy. *Am J Med* 129(11), 1198-1204. doi: 10.1016/j.amjmed.2016.05.041.

Basaran, O., Dogan, V., Beton, O., Tekinalp, M., Aykan, A.C., Kalaycioglu, E., et al. (2016). Suboptimal use of non-vitamin K antagonist oral anticoagulants: Results from the RAMSES study. *Medicine (Baltimore)* 95(35), e4672. doi: 10.1097/md.0000000000004672.

Bell, A.D., Gross, P., Heffernan, M., Deschaintre, Y., Roux, J.F., Purdham, D.M., et al. (2016). Appropriate Use of Antithrombotic Medication in Canadian Patients With Nonvalvular Atrial Fibrillation. *Am J Cardiol* 117(7), 1107-1111. doi: 10.1016/j.amjcard.2015.12.055.

Briasoulis, A., Gao, Y., Inampudi, C., Alvarez, P., Asleh, R., Chrischilles, E., et al. (2020). Characteristics and outcomes in patients with atrial fibrillation receiving direct oral anticoagulants in off-label doses. *BMC Cardiovasc Disord* 20(1), 42. doi: 10.1055/a-0952-6385

10.1186/s12872-020-01340-4.

Bruneau, A., Schwab, C., Anfosso, M., Fernandez, C., and Hindlet, P. (2019). Burden of Inappropriate Prescription of Direct Oral Anticoagulants at Hospital Admission and Discharge in the Elderly: A Prospective Observational Multicenter Study. *Drugs Aging* 36(11), 1047-1055. doi: 10.1007/s40266-019-00710-8.

Buchholz, A., Ueberham, L., Gorczynska, K., Dinov, B., Hilbert, S., Dagres, N., et al. (2018). Initial apixaban dosing in patients with atrial fibrillation. *Clin Cardiol* 41(5), 671-676. doi: 10.1002/clc.22949.

Cainzos-Achirica, M., Varas-Lorenzo, C., and Pottegard, A. (2018). Methodological challenges when evaluating potential off-label prescribing of drugs using electronic health care databases: A case study of dabigatran etexilate in Europe. 27(7), 713-723. doi: 10.1002/pds.4416.

Chan, Y.H., See, L.C., Tu, H.T., Yeh, Y.H., Chang, S.H., Wu, L.S., et al. (2018). Efficacy and Safety of Apixaban, Dabigatran, Rivaroxaban, and Warfarin in Asians With Nonvalvular Atrial Fibrillation. *J Am Heart Assoc* 7(8). doi: 10.1161/jaha.117.008150.

Chen, Y.T., and Lin, H.J. (2018). Renal Function Estimates and Dosing of Direct Oral Anticoagulants in Stroke Patients with Atrial Fibrillation: An Observational Study. *Acta Neurol Taiwan* 27(2)**,** 39-44.

Cheng, W.H., Chao, T.F., Lin, Y.J., Chang, S.L., Lo, L.W., Hu, Y.F., et al. (2019). Low-Dose Rivaroxaban and Risks of Adverse Events in Patients With Atrial Fibrillation. *Stroke* 50(9)**,** 2574-2577. doi: 10.1002/joa3.12184

10.1161/strokeaha.119.025623.

Chowdhry, U., Jacques, A., Karovitch, A., Giguere, P., and Nguyen, M.L. (2016). Appropriateness of Dabigatran and Rivaroxaban Prescribing for Hospital Inpatients. *Can J Hosp Pharm* 69(3)**,** 194-201. doi: 10.4212/cjhp.v69i3.1555.

Connolly, S.J., Ezekowitz, M.D., Yusuf, S., Eikelboom, J., Oldgren, J., Parekh, A., et al. (2009). Dabigatran versus warfarin in patients with atrial fibrillation. *N Engl J Med* 361(12)**,** 1139-1151. doi: 10.1056/NEJMoa0905561.

De Caterina, R., Kelly, P., Monteiro, P., Deharo, J.C., de Asmundis, C., Lopez-de-Sa, E., et al. (2019). Characteristics of patients initiated on edoxaban in Europe: baseline data from edoxaban treatment in routine clinical practice for patients with atrial fibrillation (AF) in Europe (ETNA-AF-Europe). 19(1)**,** 165. doi: 10.1186/s12872-019-1144-x.

Draper, E., Parkhurst, B., Carley, B., Krueger, K., Larson, T., and Griesbach, S. (2017). Comparison of Prescribing Practices with Direct Acting Oral Anticoagulant Protocols. *Am J Cardiovasc Drugs* 17(6)**,** 475-479. doi: 10.1007/s40256-017-0243-2.

Eschler, C.M., Woitok, B.K., Funk, G.C., Walter, P., Maier, V., Exadaktylos, A.K., et al. (2019). Oral Anticoagulation in Patients in the Emergency Department: High Rates of Off-Label Doses, No Difference in Bleeding Rates. *Am J Med*. doi: 10.1016/j.amjmed.2019.09.026.

Falissard, B., Picard, F., Mahe, I., Hanon, O., Touze, E., Danchin, N., et al. (2019). Apixaban for prevention of stroke and systemic embolism in patients with non-valvular atrial fibrillation in France: The PAROS cross-sectional study of routine clinical practice. *JAMA Cardiol* 112(6-7)**,** 400-409. doi: 10.1001/jamacardio.2019.1049

10.1016/j.acvd.2019.02.003.

Galaune, V., Gumbreviciute, M., and Gustafson, W.L. (2019). Real-world study of direct oral anticoagulant dosing patterns in patients with atrial fibrillation. *Medicina (Kaunas)* 17(4)**,** 1709. doi: 10.3390/medicina56010013.

Garcia Rodriguez, L.A., Martin-Perez, M., Vora, P., Roberts, L., Balabanova, Y., Brobert, G., et al. (2019). Appropriateness of initial dose of non-vitamin K antagonist oral anticoagulants in patients with non-valvular atrial fibrillation in the UK. *Intern Med J* 9(9)**,** e031341. doi: 10.1111/imj.14640

10.1136/bmjopen-2019-031341.

Gibson, C.M., Smith, C.B., Davis, S., and Scalese, M.J. (2018). Assessment of Apixaban Prescribing Patterns for Nonvalvular Atrial Fibrillation in Hospitalized Patients. *Ann Pharmacother* 52(1)**,** 54-59. doi: 10.1177/1060028017726795.

Hirsh Raccah, B., Rottenstreich, A., Zacks, N., Matok, I., Danenberg, H.D., Pollak, A., et al. (2019). Appropriateness of direct oral anticoagulant dosing and its relation to drug levels in atrial fibrillation patients. 47(4)**,** 550-557. doi: 10.1161/circresaha.118.313155

10.1007/s11239-019-01815-y.

Howerton, M.A., Suhrie, E.M., Gennari, A.S., Jones, N., and Ruby, C.M. (2019). Evaluation of Direct Oral Anticoagulant Dosing and Monitoring in Two Geriatric Outpatient Clinics. *Sr Care Pharm* 34(3)**,** 192-205.

Hussain, S., Gebran, N., Hussain, K., and Soliman, K. (2013). Drug use evaluation of dabigatran in a tertiary care hospital in United Arab Emirates. *European Journal of Hospital Pharmacy* 20(2)**,** 106-109. doi: 10.1136/ejhpharm-2012-000169.

Ikeda, T., Ogawa, S., Kitazono, T., Nakagawara, J., Minematsu, K., Miyamoto, S., et al. (2019). Outcomes associated with under-dosing of rivaroxaban for management of non-valvular atrial fibrillation in real-world Japanese clinical settings. *J Thromb Thrombolysis* 48(4)**,** 653-660. doi: 10.1007/s11239-019-01934-6.

Inoue, H., Umeyama, M., Yamada, T., Hashimoto, H., Komoto, A., and Yasaka, M. (2020). Safety and effectiveness of reduced-dose apixaban in Japanese patients with nonvalvular atrial fibrillation in clinical practice: A sub-analysis of the STANDARD study. *J Cardiol* 75(2)**,** 208-215. doi: 10.1016/j.jjcc.2019.07.007.

Jacobs, M.S., van Hulst, M., Campmans, Z., and Tieleman, R.G. (2019). Inappropriate non-vitamin K antagonist oral anticoagulants prescriptions: be cautious with dose reductions. *Neth Heart J* 27(7-8)**,** 371-377. doi: 10.1007/s12471-019-1267-9.

Jones, A.E., Witt, D.M., and Yagi, N. (2020). Creatinine clearance and inappropriate dose of rivaroxaban in Japanese patients with non-valvular atrial fibrillation. *J Thromb Thrombolysis* 35(1)**,** 110-117. doi: 10.1007/s11239-019-01904-y

10.1007/s00380-019-01457-3.

Kakkar, A.K., Mueller, I., Bassand, J.P., Fitzmaurice, D.A., Goldhaber, S.Z., Goto, S., et al. (2013). Risk profiles and antithrombotic treatment of patients newly diagnosed with atrial fibrillation at risk of

stroke: perspectives from the international, observational, prospective GARFIELD registry. *PLoS One* 8(5)**,** e63479. doi: 10.1371/journal.pone.0063479.

Kartas, A., Samaras, A., Vasdeki, D., Dividis, G., Fotos, G., Paschou, E., et al. (2019). Flaws in Anticoagulation Strategies in Patients With Atrial Fibrillation at Hospital Discharge. *J Cardiovasc Pharmacol Ther* 24(3)**,** 225-232. doi: 10.1177/1074248418821712.

Kato, Y., Hayashi, T., Tanahashi, N., and Takao, M. (2018). The Dose of Direct Oral Anticoagulants and Stroke Severity in Patients with Acute Ischemic Stroke and Nonvalvular Atrial Fibrillation. *J Stroke Cerebrovasc Dis* 27(6)**,** 1490-1496. doi: 10.1016/j.jstrokecerebrovasdis.2017.12.038.

Khan, F., Huang, H., and Datta, Y.H. (2016). Direct oral anticoagulant use and the incidence of bleeding in the very elderly with atrial fibrillation. *J Thromb Thrombolysis* 42(4)**,** 573-578. doi: 10.1007/s11239-016-1410-z.

Kim, J.Y., Kim, S.H., Myong, J.P., Kim, Y.R., Kim, T.S., Kim, J.H., et al. (2019). Outcomes of Direct Oral Anticoagulants in Patients With Mitral Stenosis. *PLoS One* 73(10)**,** 1123-1131. doi: 10.1371/journal.pone.0213517

10.1016/j.jacc.2018.12.047.

Lavoie, K., Turgeon, M.H., Brais, C., Larochelle, J., Blais, L., Farand, P., et al. (2016). Inappropriate dosing of direct oral anticoagulants in patients with atrial fibrillation. *J Atr Fibrillation* 9(4)**,** 1478. doi: 10.4022/jafib.1478.

Lee, K.N., and Choi, J.I. (2020). Effectiveness and Safety of Off-label Dosing of Non-vitamin K Antagonist Anticoagulant for Atrial Fibrillation in Asian Patients. 10(1)**,** 1801. doi: 10.1038/s41598-020-58665-5.

Lee, S.R., Choi, E.K., Han, K.D., Jung, J.H., Oh, S., and Lip, G.Y.H. (2019). Optimal Rivaroxaban Dose in Asian Patients With Atrial Fibrillation and Normal or Mildly Impaired Renal Function. *Stroke* 50(5)**,** 1140-1148. doi: 10.1161/strokeaha.118.024210.

Lee, S.R., and Lee, Y.S. (2019). Label Adherence for Non-Vitamin K Antagonist Oral Anticoagulants in a Prospective Cohort of Asian Patients with Atrial Fibrillation. 60(3)**,** 277-284. doi: 10.3349/ymj.2019.60.3.277.

Leef, G.C., Perino, A.C., Askari, M., Fan, J., Ho, P.M., Olivier, C.B., et al. (2019). Appropriateness of Direct Oral Anticoagulant Dosing in Patients With Atrial Fibrillation: Insights From the Veterans Health Administration. *J Pharm Pract***,** 897190019828270. doi: 10.5603/KP.a2019.0033

10.1177/0897190019828270.

McAlister, F.A., Garrison, S., Kosowan, L., Ezekowitz, J.A., and Singer, A. (2018). Use of Direct Oral Anticoagulants in Canadian Primary Care Practice 2010-2015: A Cohort Study From the Canadian

Primary Care Sentinel Surveillance Network. *J Am Heart Assoc* 7(3). doi: 10.1161/jaha.117.007603.

Morris, J.K., MacCallum, P., Arbel, R., Sergienko, R., Hammerman, A., Greenberg-Dotan, S., et al. (2019). Effectiveness and Safety of Off-Label Dose-Reduced Direct Oral Anticoagulants in Atrial Fibrillation. *Br J Haematol* 132(7)**,** 847-855.e843. doi: 10.1111/bjh.15808

10.1016/j.amjmed.2019.01.025.

Moudallel, S., Steurbaut, S., Cornu, P., and Dupont, A. (2018). Appropriateness of DOAC Prescribing Before and During Hospital Admission and Analysis of Determinants for Inappropriate Prescribing. *Front Pharmacol* 9**,** 1220. doi: 10.3389/fphar.2018.01220.

Murata, N., Okumura, Y., Yokoyama, K., Matsumoto, N., Tachibana, E., Kuronuma, K., et al. (2019). Clinical Outcomes of Off-Label Dosing of Direct Oral Anticoagulant Therapy Among Japanese Patients With Atrial Fibrillation Identified From the SAKURA AF Registry. *Circ J* 83(4)**,** 727-735. doi: 10.2174/1570161117666190206230516

10.1253/circj.CJ-18-0991.

Navarro-Almenzar, B., Cerezo-Manchado, J.J., Caro-Martinez, C., Garcia-Candel, F., Flores Blanco, P.J., Ruiz, G.E., et al. (2019). Real-life behaviour of direct oral anticoagulants in a Spanish cohort with non-valvular atrial fibrillation: Refase Registry. *J Cardiol* 35(12)**,** 2035-2041. doi: 10.1016/j.jjcc.2019.06.002

10.1080/03007995.2019.1647735.

Okumura, Y., Yokoyama, K., Matsumoto, N., Tachibana, E., Kuronuma, K., Oiwa, K., et al. (2017). Current use of direct oral anticoagulants for atrial fibrillation in Japan: Findings from the SAKURA AF Registry. *Journal of Arrhythmia* 33(4)**,** 289-296. doi: 10.1016/j.joa.2016.11.003.

Paciaroni, M., Agnelli, G., Caso, V., Silvestrelli, G., Seiffge, D.J., Engelter, S., et al. (2019). Causes and Risk Factors of Cerebral Ischemic Events in Patients With Atrial Fibrillation Treated With Non-Vitamin K Antagonist Oral Anticoagulants for Stroke Prevention. *Stroke* 50(8)**,** 2168-2174. doi: 10.1161/strokeaha.119.025350.

Patel, M.R., Mahaffey, K.W., Garg, J., Pan, G., Singer, D.E., Hacke, W., et al. (2011). Rivaroxaban versus warfarin in nonvalvular atrial fibrillation. *N Engl J Med* 365(10)**,** 883-891. doi: 10.1056/NEJMoa1009638.

Pisters, R., van Vugt, S.P.G., Brouwer, M.A., Elvan, A., Ten Holt, W.L., Zwart, P.A.G., et al. (2017). Real-life use of Rivaroxaban in the Netherlands: data from the Xarelto for Prevention of Stroke in Patients with Atrial Fibrillation (XANTUS) registry. *Neth Heart J* 25(10)**,** 551-558. doi: 10.1007/s12471-017-1009-9.

Sato, T., Aizawa, Y., Fuse, K., Fujita, S., Ikeda, Y., Kitazawa, H., et al. (2018). The Comparison of Inappropriate-Low-Doses Use among 4 Direct Oral Anticoagulants in Patients with Atrial Fibrillation: From the Database of a Single-Center Registry. *J Stroke Cerebrovasc Dis* 27(11)**,** 3280-3288. doi: 10.1016/j.jstrokecerebrovasdis.2018.07.028.

Saunders, J.A., Gustafson, W.L., and Vazquez, S.R. (2019). Real-world assessment of off-label direct oral anticoagulant dosing for venous thromboembolism. 48(3)**,** 506-510. doi: 10.1007/s11239-019-01904-y.

Schwartz, J., Merrill, S., de Leon, N., Thompson, A., and Fang, M. (2017). Dosing Accuracy of Direct Oral Anticoagulants in an Academic Medical Center. *Clin Cardiol* 12(7)**,** 544-550. doi: 10.1002/clc.22746

10.12788/jhm.2769.

Shrestha, S., Baser, O., and Kwong, W.J. (2018). Effect of Renal Function on Dosing of Non-Vitamin K Antagonist Direct Oral Anticoagulants Among Patients With Nonvalvular Atrial Fibrillation. *Ann Pharmacother* 52(2)**,** 147-153. doi: 10.1177/1060028017728295.

Sieg, A., and Nappi, J. (2015). Evaluation of dosing practices of rivaroxaban and dabigatran. *Journal of Pharmacy Technology* 31(4)**,** 149-154. doi: 10.1177/8755122514567923.

Steinberg, B.A., Shrader, P., Thomas, L., Ansell, J., Fonarow, G.C., Gersh, B.J., et al. (2016). Off-Label Dosing of Non-Vitamin K Antagonist Oral Anticoagulants and Adverse Outcomes: The ORBIT-AF II Registry. *J Am Coll Cardiol* 68(24)**,** 2597-2604. doi: 10.1016/j.jacc.2016.09.966.

Suwa, M., Morii, I., and Kino, M. (2019). Rivaroxaban or Apixaban for Non-Valvular Atrial Fibrillation-Efficacy and Safety of Off-Label Under-Dosing According to Plasma Concentration. *Circ J* 83(5)**,** 991-999. doi: 10.1253/circj.CJ-18-1282.

Tellor, K.B., Patel, S., Armbruster, A.L., and Daly, M.W. (2015). Evaluation of the appropriateness of dosing, indication and safety of rivaroxaban in a community hospital. *J Clin Pharm Ther* 40(4)**,** 447-451. doi: 10.1111/jcpt.12288.

Tellor, K.B., Wang, M., Green, M.S., and Armbruster, A.L. (2017). Evaluation of Apixaban for the Treatment of Nonvalvular Atrial Fibrillation with Regard to Dosing and Safety in a Community Hospital. *Journal of Pharmacy Technology* 33(4)**,** 140-145. doi: 10.1177/8755122517706423.

Umei, M., Kishi, M., Sato, T., Shindo, A., Toyoda, M., Yokoyama, M., et al. (2017). Indications for suboptimal low-dose direct oral anticoagulants for non-valvular atrial fibrillation patients. *J Arrhythm* 33(5)**,** 475-482. doi: 10.1016/j.joa.2017.05.008.

Vinter, N., Linder, M., Andersen, M., Pedersen, A.B., Madsen, M., Schachterle, S.E., et al. (2019). Classification and characteristics of on-label and off-label apixaban use in Denmark and Sweden. *Pharmacoepidemiol Drug Saf* 28(6)**,** 867-878. doi: 10.1002/pds.4778.

Viprey, M., Jeannin, R., Piriou, V., Chevalier, P., Michel, C., Aulagner, G., et al. 2016. Prevalence of drug-related problems associated with direct oral anticoagulants in hospitalized patients: a multicenter, cross-sectional study. *Journal of clinical pharmacy and therapeutics* [Online], (no pagination). Available: https://www.cochranelibrary.com/central/doi/10.1002/central/CN-01291557/full.

Yamaji, H., Murakami, T., Hina, K., Higashiya, S., Kawamura, H., Murakami, M., et al. (2017). Safety and Efficacy of Underdosing Non-vitamin K Antagonist Oral Anticoagulants in Patients Undergoing Catheter Ablation for Atrial Fibrillation. *J Cardiovasc Pharmacol* 69(2)**,** 118-126. doi: 10.1097/fjc.0000000000000448.

Yao, X., Shah, N.D., Sangaralingham, L.R., Gersh, B.J., and Noseworthy, P.A. (2017). Non-Vitamin K Antagonist Oral Anticoagulant Dosing in Patients With Atrial Fibrillation and Renal Dysfunction. *J Am Coll Cardiol* 69(23)**,** 2779-2790. doi: 10.1016/j.jacc.2017.03.600.

Yiginer, O., Tezcan, M., Erdal, E., Degirmencioglu, G., Acar, G., Ergelen, M., et al. (2017). A real-world, retrospective, observational study of dabigatran and rivaroxaban in turkey: Elderly patients receive inappropriately low dose of rivaroxaban. *International Journal of Clinical and Experimental Medicine* 10(7)**,** 10634-10642.
